# Evidence for temporal population replacement and the signature of ecological adaptation in a major Neotropical malaria vector in Amazonian Peru

**DOI:** 10.1186/s12936-015-0863-4

**Published:** 2015-09-29

**Authors:** William Lainhart, Sara A. Bickersmith, Kyle J. Nadler, Marta Moreno, Marlon P. Saavedra, Virginia M. Chu, Paulo E. Ribolla, Joseph M. Vinetz, Jan E. Conn

**Affiliations:** Department of Biomedical Sciences, School of Public Health, State University of New York at Albany, Albany, NY USA; Wadsworth Center, New York State Department of Health, Griffin Laboratory, 5669 State Farm Road, Building 1, Room 101, Slingerlands, NY 12159 USA; Division of Infectious Diseases, Department of Medicine, University of California, San Diego, La Jolla, CA USA; Asociación Benéfica PRISMA, Iquitos, Peru; Universidade Estadual Paulista, São Paulo, Brazil; Instituto de Medicine Tropical “Alexander von Humboldt”, Universidad Peruana Cayetano Heredia, Lima, Peru

**Keywords:** Population replacement, Ecological adaptation, *Anopheles darlingi*, Malaria, Human biting rate, Microsatellites

## Abstract

**Background:**

The major Neotropical malaria vector, *Anopheles darlingi*, was reintroduced into the Iquitos, Loreto, Peru area during the early 1990s, where it displaced other anophelines and caused a major malaria epidemic. Since then, case numbers in Loreto have fluctuated, but annual increases have been reported since 2012.

**Methods:**

The population genetic structure of *An. darlingi* sampled before and after the introduction of long-lasting insecticidal nets (LLINs) was investigated to test the hypothesis of temporal population change (2006 vs. 2012). Current samples of *An. darlingi* were used to test the hypothesis of ecological adaptation to human modified (highway) compared with wild (riverine) habitat, linked to forest cover. In total, 693 *An. darlingi* from nine localities in Loreto, Peru area were genotyped using 13 microsatellite loci. To test the hypothesis of habitat differentiation in *An. darlingi* biting time patterns, HBR and EIR, four collections of *An. darlingi* from five localities (two riverine and three highway) were analysed.

**Results:**

Analyses of microsatellite loci from seven (2006) and nine settlements (2012–2014) in the Iquitos area detected two distinctive populations with little overlap, although it is unclear whether this population replacement event is associated with LLIN distribution or climate. Within the 2012–2014 population two admixed subpopulations, A and B, were differentiated by habitat, with B significantly overrepresented in highway, and both in near-equal proportions in riverine. Both subpopulations had a signature of expansion and there was moderate genetic differentiation between them. Habitat and forest cover level had significant effects on HBR, such that *Plasmodium* transmission risk, as measured by EIR, in peridomestic riverine settlements was threefold higher than in peridomestic highway settlements. HBR was directly associated with available host biomass rather than forest cover.

**Conclusions:**

A population replacement event occurred between 2006 and 2012–2014, concurrently with LLIN distribution and a moderate El Niño event, and prior to an increase in malaria incidence. The likely drivers of this replacement cannot be determined with current data. The present-day *An. darlingi* population is composed of two highly admixed subpopulations, which appear to be in an early stage of differentiation, triggered by anthropogenic alterations to local habitat.

**Electronic supplementary material:**

The online version of this article (doi:10.1186/s12936-015-0863-4) contains supplementary material, which is available to authorized users.

## Background

The replacement of a disease vector or an insect agricultural pest with a more benign strain has been a topic of intense research since the late twentieth century [1–4]. Currently, research focused on the control and replacement of *Anopheles* populations utilizes sterile insect techniques and the creation of transgenic mosquitoes with reduced competence for *Plasmodium* [5–9]. However, replacement and genetic turnover events do occur naturally, and may be fairly common, given the pressures of changing and altered habitats and environments, as well as influence imposed directly by humans, through insecticide and pesticide application. A recent, documented example of such an event, detected using microsatellites, occurred in the agricultural pest *Bemisia tabaci* (silverleaf whitefly) in Queensland, Australia. Over a 3-month period, between 2006 and 2007, the most abundant subpopulation of the silverleaf whitefly was nearly completely replaced by a much less abundant one, though the reasons for this replacement are unclear [10]. In New Jersey, over a 9 year period, *Aedes albopictus* and *Aedes japonicus* invaded and partially displaced *Aedes triseriatus* [11]. During this time, the abundance of both *Ae. albopictus* and *Ae. japonicus* doubled, while *Ae. triseriatus* abundance decreased by a factor of three [11]. Although *Ae. triseriatus* is a known arboviral vector [12], *Ae. albopictus* and *Ae. japonicus* have been shown to be much more competent vectors of a wide range of arboviruses, including chikungunya and dengue [13–16]. Additionally, species replacement was observed in anophelines of the Brazilian Amazon (Amazonas state), between August 2008 to March 2010. The authors observed the replacement of *Anopheles darlingi* with *Anopheles albitarsis s.l.* following distribution of long-lasting insecticidal nets (LLINs) and increased indoor residual spraying (IRS), that resulted in a decrease in malaria incidence rates in the study localities [17].

*Anopheles darlingi* was first collected in Peru in 1933, near the Brazilian border of Loreto [18]. Later surveys (1953–1957) conducted by the Ministry of Health detected *An. darlingi* in localities across Loreto and in one in Cusco department, southern Peru [19]. An intense anti-malaria and insecticide application campaign in 1957 was quite effective, with malaria nearly eliminated in some Peruvian departments within a year [20], and reached a low of 1500 cases in 1965, down from approximately 95,000 in 1944 [21]. In 1965–1966, a survey of anophelines along the Yavarí river (Brazilian border of Loreto) found *An. darlingi* to be the most abundant species (87 % of 4392 anophelines collected) [22]. Although *An. darlingi* was still detected in the Yavarí river watershed in 1971 collections, it was absent in the peri-Iquitos area [23]. In a separate study, *Anopheles benarrochi* was the most abundant anopheline (98 % of those collected, which included *Anopheles oswaldoi*, *Anopheles mattogrossensis*, and *Anopheles rangeli*) in two riverine localities in the Peruvian Amazon [24].

By the late 1980s, DDT insecticide spraying ended in Loreto [21, 25, 26], in part because no malaria cases were detected in 1988 [21]. Although *An. darlingi* was not detected in Iquitos in 1989 [27] or in the peri-Iquitos area in 1991 [28], by 1996 it had re-emerged in the Iquitos area concomitantly with a malaria epidemic [21, 29–32]. In the early 1990s, *An. darlingi* had apparently displaced other anophelines, such as *An. benarrochi* [22, 24], *An. oswaldoi* [24] and *An. mattogrossensis* [28], establishing itself as the most abundant regional anopheline, and the primary malaria vector in several river basins [33–35], i.e., the number of malaria cases rose to over 150,000 in 1997 [21]. Since 2000, case numbers in Loreto have fluctuated from 22,406 (39.6 % of cases in Peru) in 2000 to 60,186 (93.7 % of cases) in 2014 [36]. The reason(s) for such a dramatic change remain unclear and have not been tested.

Evolutionary diversification is thought to be driven by ecological speciation, which occurs when populations adapt to distinctive environments or habitats, ultimately leading to reproductive isolation [37, 38]. The remarkably broad distribution of *An. darlingi* from southern Mexico to northern Argentina [39], for years has begged the question of why multiple species have not been detected [40], but see Emerson et al. [41], as they have in *An. albitarsiss.l.*, comprised of nine species, with a similar overall distribution as *An. darlingi* [42, 43]. One hypothesis is that a predominantly riverine habitat in *An*. *darlingi* maintains patterns of population connectivity across diverse biogeographic regions; whereas the shallow sunlit pools where members of *An. albitarsiss.l.* breed [44, 45] may lead to disruption in patterns of gene flow among populations, culminating in speciation. Evidence for diversification in *An. darlingi* is mixed. Despite significant differentiation between identified genotypes of *An. darlingi* using microsatellites (genotype 1—Amazonia, genotype 2—Central America, pairwise *F*_*ST*_ range 0.1859–0.3901) [46], analysis of two complete *An. darlingi* mtDNA genomes representing these genotypes supported monotypic species status [47]. Notwithstanding the mtDNA results, a recent study using Restriction site Associated DNA Sequencing (RAD-seq) hypothesizes the existence of three putative species within Brazil, associated with major biogeographical regions [41]. Genetic differentiation within *An. darlingi* has been explained by geographic barriers and/or isolation by distance [46, 48, 49]; the standing genetic diversity within the species is thought to support phenotypic plasticity in habitat, anthropophagy, exo/endophily, and biting times.

Population genetic studies of *An. darlingi* in Peru detected the presence of single, panmictic populations with limited to moderate diversity, using AFLPs [50] or microsatellites [46]. *An. darlingi* is often referred to as a riverine species [51–53], though an association exists between larval habitats and anthropogenically altered sites, such as fish ponds and pools left by gold-mining and road construction [54, 55]. Two seasonally distinct subpopulations of *An. darlingi*, were recently discovered along the Madeira River in Rondônia, Brazil, that may provide evidence of adaptation to human-altered habitat [56].

In the Iquitos region, Vittor et al. [25] detected a statistically significant association between forest cover percentage and *An. darlingi* human biting rate (HBR). The experimental design, which focused on communities along the Iquitos-Nauta highway, found that HBR (from 18:00 to 24:00 h) was highest in peridomestic collections (6.5 bites per person in 0–20 % forest cover) and decreased with increasing forest cover percentage [25]. A more recent survey of communities along the Mazan river, north of Iquitos, found much higher *An. darlingi* HBRs ranging from 0.102 to 41.13 bites per person per hour, with entomological inoculation rates (EIRs) as high as 5.3 infective bites per person per night in mostly pristine riverside camps used by local fisherman and loggers [34].

The present study extended the HBR studies by Vittor et al. [25] and the population genetics results by Mirabello et al. [46], using *An. darlingi* collected in highway and riverine habitats, at three levels of forest cover, in the peri-Iquitos region. The following questions were considered: (1) is there a signature of temporal change in *An. darlingi* populations collected prior to the PAMAFRO LLIN intervention [46] and those collected after the intervention? (2) Were there regional climatic events that might confound such a simple explanation, as in Suriname [57]? (3) Are there genetic differences between riverine and highway populations of *An. darlingi*? (4) Do the vector biology metrics of HBR and EIR, and risk of *Plasmodium* infection differ in riverine compared with highway communities? and (5) Is there a direct influence of forest cover level on vector biology metrics?

## Methods

### Mosquito collections

Adult female *An. darlingi* specimens were collected using human landing catch (HLC) with two collectors in 12-h peridomestic collections in nine localities [San José de Lupuna (LUP), Santo Tomás (STO), Villa Buen Pastor (VBP), El Dorado (DOR), Nuevo Horizonte (NHO), El Triunfo (TRI), Cahuide (CAH), Nuevo Progreso (NPR) and Santa Emilia (SEM)], and identified morphologically [58–60].

Six localities (DOR, NHO, TRI, CAH, NPR and SEM), denoted herein as “deforestation study sites”, included collections in *chacra* (community garden) and forest locations. An Additional file lists the collection and locality information (see Additional file 1). Adult *An. darlingi* specimens were bisected and the head/thorax of each was sent to the Conn laboratory at the Wadsworth Center (New York State Department of Health) for DNA extraction, genotyping and *Plasmodium* infection determination. This study was approved by the Human Subjects Protection Program of the University of California San Diego, La Jolla, and by the Comité de Ética of the Universidad Peruana Cayetano Heredia and Asociación Benéfica PRISMA, Lima, Peru. The New York State IRB considers HLC to be an occupational health/risk management issue, and malaria prophylaxis drugs were offered to collectors in accordance with this policy.

### Riverine versus highway habitat

**Fig. 1:**
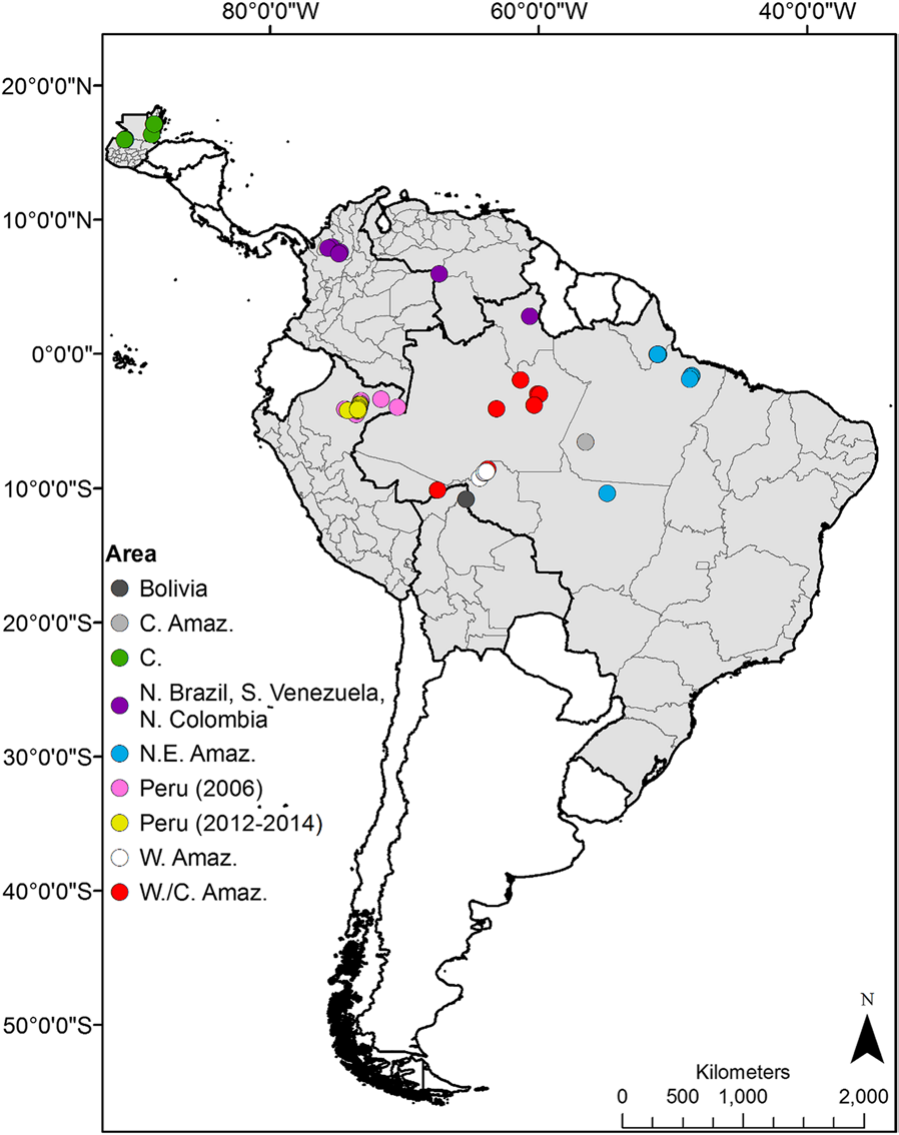
Map of Central/South America showing locations of collected *Anopheles darlingi* used in these analyses (Additional file 1). Countries shaded *light grey* are those represented in the study sample. Collection sites are *colored* to represent subpopulation structure, as seen in Fig. 2. Shapefiles for administrative boundaries of Central and South American countries obtained from [122]. *C* Central, *NE* Northeastern, *Amaz* Amazonian, *W* Western, *N* Northern, *S* Southern

The study area is in the Loreto department of Amazonian Peru, near the city of Iquitos (3.74°S, 73.25°W). Iquitos, with a population of 406,340 people [61], lies at the intersection of three rivers: the Amazon, the Nanay and the Itaya. *Anopheles darlingi* specimens were collected in localities situated along the nearby rivers and/or the Iquitos-Nauta highway (Fig. 1).

Vittor et al. [25] reported an association between forest cover level (deforestation) and *An. darlingi* HBR, avoiding collection of *An. darlingi* in the riverine area around the intersection of the Itaya River and the Iquitos-Nauta highway. In the present study, the effects of habitat (riverine vs. highway) on *An. darlingi* population genetics, HBR and EIR were analysed, and compared with those of Vittor et al. [25]. Habitat assignment depended on proximity of the settlement to the nearest river: riverine localities were <1 km and highway localities >2 km from the nearest river. After habitat assignment, all nineteen Bioclim variables [62] were used to assess differences between habitats, to ensure the biological relevance of this assignment, using Student’s t-test (α = 0.10).

### Determination of forest cover level in highway and riverine communities

In each locality, a peridomestic collection site was chosen within 10 m of a home, near the center of the village (visually estimated forest cover level <20 %). *Chacra* and forest sites, in the deforestation study, were at least 1 km from the peridomestic site, and at least 1 km from each other. In two remote localities (NPR and SEM), *chacra* were very near settlements, so distance criteria were modified accordingly. *Chacra* sites were chosen as an intermediate forest cover level area (visually estimated forest cover 20–60 %) where HBR was expected to be lower than peridomestic and higher than forest sites [25]. These sites were initially deforested for agriculture and are now primarily gardens (banana, manioc other crops), or dense shrubs and high grass. Forest sites were chosen as high forest cover level areas within dense rainforest (visually estimated forest cover >60 %) where low HBRs, and little, if any, malaria transmission was expected because of a lack of breeding site types preferred by *An*. *darlingi* (low acidity of water bodies and partial shading) [63].

### Ground truthing and hemispherical imaging

Forest cover level near each collection site (3 in each of the six deforestation villages noted above) was estimated using two different methods: satellite and digital hemispherical imagery. GeoEye-1 satellite imagery, 4-bands at 50 cm resolution, was obtained between September 2013 and August 2014 (LandInfo Worldwide Mapping LLC, Highlands Ranch, CO, USA). Thirty random GPS coordinates were selected within 100 m of each peridomestic and *chacra* mosquito collection site using ArcGIS v.10.2.2 (ESRI, Redlands, CA, USA). Within the forest, only the collection site was visited for ground-truthing, due to the very high forest cover and safety concerns. The points were chosen using the “Create Random Points” tool of the Spatial Analyst Extension. Each of these sites was visited and pertinent information was collected, including ground type (i.e., grass, asphalt, bare ground) and forest cover was visually estimated. To determine the percentage of forest within 50 and 100 m of each collection site, satellite imagery was analysed using ArcGIS v.10.2.2 and GIMP v.2.8.10 image editing software [64]. Images were analysed by manually selecting areas without forest cover, such as bare ground, shrubs, grass, homes or water, removing them, and filling that space with black. The percentage of non-black pixels within the image was then determined using the Color > Info > Histogram tool—all pixels not coloured pure black were considered to be forested (see Additional file 2).

Additionally, at each ground-truthing site, hemispherical images were taken. Digital hemispherical photography can estimate the level of forest canopy [65, 66] using a digital camera with a fisheye lens mounted on a tripod, pointing directly upward. An additional image was taken directly north and parallel to the ground, so the site could be characterized appropriately. Each photograph was taken at the same height relative to the ground (approximately 1.36 m). The resulting photographs were analysed using CAN-EYE v.6.314 [67]. These images were analysed as “hemispherical images” and “upward” facing. Each image was individually masked so that humans or man-made objects (homes, towers, power lines, etc.) would not be counted as forest canopy. Images were batch analysed (up to 8 images at a time) and an average forest canopy cover (percentage) was reported by the programme per batch. For each collection site, the overall average canopy cover was calculated (see Additional file 3).

Forest cover in the area surrounding mosquito collection sites was calculated, post hoc, using three methods: satellite imagery analysis at 50 m (SI50) and 100 m (SI100) radii, and hemispherical photography (HP) at 30 randomly chosen sites within a 100 m radius (see Additional files 2, 3).

### Genotyping and analysis

Fourteen microsatellite loci [56, 68, 69] (see Additional file 4) were PCR amplified using fluorescently labelled primers, for each of the *An. darlingi* specimens collected for genotyping. PCR reactions were 20 µL containing 1× PCR buffer with 1.5 mM MgCl_2_ (Qiagen), 0.2 mM dNTPs, 0.35 µM of each primer, 0.02 U/µL *Taq* Polymerase (Qiagen), 28:1 Ab:Taq ratio of ClonTech TaqStart antibody and 1 µL of genomic DNA. Cycling conditions included an initial denaturation step of 2.5 min at 95 °C, followed by 35 cycles of denaturation at 94 °C for 30 s, annealing at 60 °C for 30 s, and elongation at 72 °C for 30 s; and a final extension step at 72 °C for 5 or 30 min, depending on microsatellite locus (see Additional file 4). Successful amplification of each sample at all loci was verified on 1 % agarose gels. PCR products were genotyped by the Applied Genomic Technologies Core at the Wadsworth Center, New York State Department of Health using an ABI3730 DNA Analyzer (Applied Biosystems) with GeneScan™ 600 LIZ^®^ dye size standard (Applied Biosystems). Data were analysed and alleles called using GeneMapper v.4.0 software (Applied Biosystems). The Microsoft Excel database of alleles was converted to file formats specific to other analysis programs using CONVERT v.1.31 [70].

Initial population genetic analyses were completed comparing the current data set with those published previously [46, 56, 71–73] plus two additional localities from Bolivia and Venezuela (Fig. 1, Additional file 1), using five microsatellite loci (ADC02, ADC28, ADC110, ADC137, ADC138). In these analyses, individuals with missing data were excluded. Population structure among all genotyped *An. darlingi* was determined using Bayesian analyses implemented by STRUCTURE v.2.3.4 [74, 75]. These analyses were conducted using the admixture model without prior information regarding sampling group, with correlated allele frequencies, 500,000 burn-in and 2 million MCMC replications for 20 iterations of each K = 1–15. The result files were analysed, to determine optimal K (the K at which ΔK has the largest value), with the Evanno method implemented by STRUCTURE Harvester [76]. The resulting 20 files for optimal K were averaged, at the population level, using the LargeKGreedy method in the CLUMPP v.1.1.2 program [77]. A Cavalli-Sforza chord distance [78], neighbour joining consensus tree, representing genetic differentiation among populations, was created using Populations v.1.2.31 [79] with 1000 bootstrap replicates. The tree was visualized with Mesquite v.3.02 [80]. Population differences in individual mosquito genotypes, by STRUCTURE subpopulation, were visualized with Factorial Correspondence Analysis (FCA) in GENETIX v.4.05.2 [81].

To test the hypothesis of ecological adaptation to human modified (highway) compared with wild (riverine) habitat, 14 microsatellite loci were analysed in the *An. darlingi* population in the peri-Iquitos area. Individuals with missing data at more than one locus were excluded. Individual *An. darlingi* were assigned to subpopulations using the 0.8 membership threshold suggested by Vähä and Primmer [82], after STRUCTURE Bayesian analysis (K = 1–5), as described above. The 20 results files for optimal K were averaged, at the individual level, as above. Subsequent population genetics analyses were conducted on mosquitoes assigned to a subpopulation, and those not assigned (i.e., admixed) were excluded. Subpopulation differences in individual mosquito genotypes were also visualized by conducting FCA [81].

The average number of alleles (*A*), and expected and observed heterozygosity (*H*_*E*_ and *H*_*O*_, respectively) over all loci, linkage disequilibrium, and deviations from Hardy–Weinberg Equilibrium (HWE) were calculated using Arlequin v.3.5 [83]. The number of private alleles over all loci was obtained using the CONVERT v.1.31 program [70]. Measures of differentiation (*F*_*ST*_), inbreeding (*F*_*IS*_), and gene flow (effective number of migrants, *N*_*M*_) were estimated using the program GENETIX [81, 84]. Non-neutral loci were identified using the program LOSITAN selection detection workbench [85, 86], then removed from the data set. This new data set was then analysed, using LOSITAN, under the Stepwise Mutation Model (SMM), 99.5 % confidence interval and “Force mean *F*_*ST*_” option to calculate the neutral *F*_*ST*_. The data set was also run under the IAM Model, and the same outlier locus was identified. “Neutral *N*_*M*_” was then calculated using the following equation: $N_{M} \approx \left[ { \left( {\frac{1}{{F_{ST} }} - 1} \right) \times 0.25} \right]$ [87]. In instances of multiple or pair-wise comparisons, the nominal significance level (α = 0.05) was adjusted by Bonferroni correction.

Samples from Mirabello et al. [46] were genotyped at an additional eight microsatellite loci, bringing the number of shared loci between those specimens and those in the current study to 13. STRUCTURE Bayesian analyses (K = 1–5) were conducted, as described above, to verify the differences between these populations. Additionally, allelic distributions, per locus, were compared between populations using the nonparametric Kolmogorov–Smirnov (KS) test for equality in continuous distribution functions in Rstudio v.0.98.1091 (Boston, MA, USA) using R v.3.1.2 [88]. Finally, STRUCTURE Bayesian analyses (K = 1–5) were conducted, as described above but with only ten iterations, to visualize the population structure of the 2006 *An. darlingi* population.

### Real-time PCR for *Plasmodium* detection

Individual *An. darlingi* heads/thoraces were extracted by hand or with a QIAcube using the DNeasy Blood & Tissue Kit (Qiagen). DNA concentration of each extraction was determined using a Qubit^®^ 2.0 fluorometer with the Qubit^®^ dsDNA high sensitivity (HS) assay. Detection of *Plasmodium* infection was conducted using real-time PCR of the small subunit of the 18S rRNA, using a triplex TaqMan assay (Life Technologies), as described in [89].

### Human biting rate and time and entomological inoculation rate

Biting time, HBR and EIR were calculated using collection information from 4 monthly, 12-h collections in five of the deforestation study sites. The collections for the calculation of these measurements were undertaken between April and June 2013, the peak malaria transmission season in Loreto [21]. Because Santa Emilia (SEM) was added to this project after it began, it was excluded from the human biting time, HBR and EIR analyses, to ensure comparability among the remaining sites. Human biting time was visualized by plotting the average (±95 % confidence intervals) hourly number of *An. darlingi* collected (18:00–06:00 h) to determine whether biting patterns differed by habitat. The patterns were statistically compared for differences using the KS test, as described above. HBR was evaluated as the average number of *An. darlingi* bites per collector per hour. Entomological inoculation rate (EIR) was calculated by multiplying the HBR by the proportion of *An. darlingi* that were determined to be *Plasmodium*-positive. The effects of habitat, forest cover and their interaction were statistically evaluated using linear modelling. These effects on EIR were not evaluated due to the small number of infected mosquitoes.

### *Anopheles darlingi* wing length measurements

Wing lengths were measured, using a stereomicroscope, for a subset of *An. darlingi* collected in the deforestation sites. Differences in mean (±95 % confidence interval) wing length were evaluated by habitat, forest cover level, and their interaction using linear modelling implemented in Rstudio v.0.98.1091, and subpopulation membership using a Student’s t-test, with a smaller subset of individuals (not all of the microsatellite genotyped individuals had available wing length measurements).

## Results

### Collection sites and information

In Loreto, the ecology at the intersection of the highway and river is unknown. Therefore, STO (peri-urban, bordering Nanay river) and CAH (intersection between Itaya River and Iquitos-Nauta highway) were individually assessed using STRUCTURE results and grouped with the habitat their results most closely matched (STO—highway; CAH—riverine). After final assignments to habitat, statistically significant differences in Bioclim variables for seasonality (Bio4 and Bio7) and extreme/limiting factors (Bio5, Bio8, Bio11, Bio13) in temperature and precipitation were observed between riverine and highway localities (*p* < 0.10). Within peridomestic collection sites, domestic animals were noted as follows: chickens, dogs, cats, cows, ducks, pigs, and turkeys. Domestic animals were detected only at a few *chacra* sites (cows and pigs), and none were detected in forest sites.

### *Anopheles darlingi* population structure

**Fig. 2:**
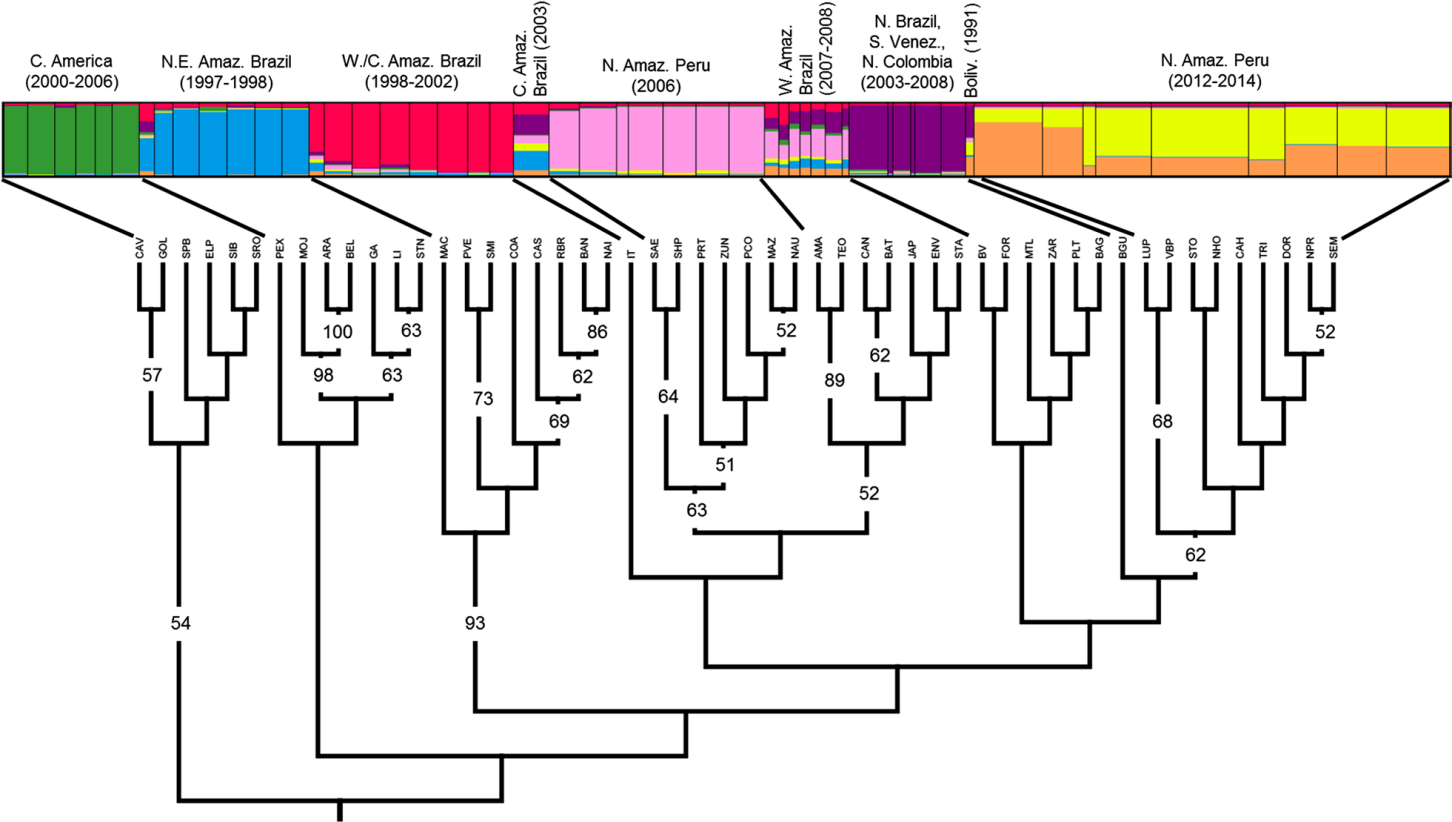
Population structure (K = 7) and Cavalli-Sforza chord distance neighbor joining tree of *Anopheles darlingi* collections in Central and South America from 1991–2014, using five microsatellite loci. Tree rooted with Central American collections. Bootstrap values greater than 50 shown. *C* Central, *NE* Northeastern, *Amaz* Amazonian, *W* Western, *N* Northern, *S* Southern, *Venez* Venezuela, *Boliv* Bolivia

**Fig. 3:**
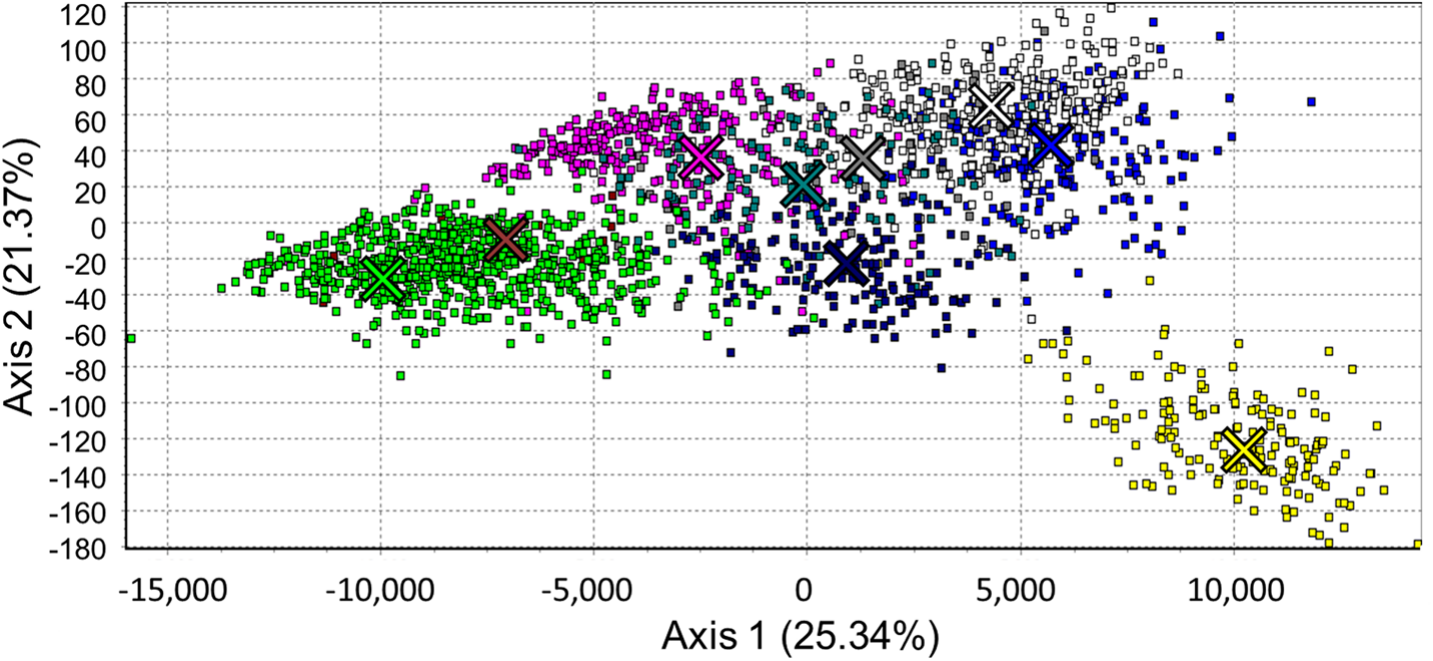
Factorial correspondence analysis of subpopulations identified in STRUCTURE analyses (Fig. 2). Axes *1* and *2* explain 25.34 and 21.37 % of the inertia in individual *Anopheles darlingi* genotypes, respectively. Each uniquely colored *X* corresponds to the centroid of each similarly colored subpopulation. *Yellow* Central American; *blue* Northeastern Amazonian Brazil; *white* Western/Central Amazonian Brazil; *grey* Central Amazonian Brazil; *pink* Peru (2006); *teal* Western Amazonian Brazil; *dark blue* Northern Brazil, Southern Venezuela and Northern Colombia; *brown* Bolivia; *lime green* Peru (2012–2014)

STRUCTURE and STRUCTURE Harvester analyses of the comparison among all samples across the Neotropics resulted in an optimal K = 2, using five microsatellite loci. When comparing this result to previous studies [46], K = 2 was not biologically meaningful. After excluding K = 2, STRUCTURE Harvester analyses resulted in an optimal K = 7 (Fig. 2). According to the STRUCTURE program manual, a K should be chosen if it represents population structure that can be explained biologically, and if some proportion of individuals are strongly assigned to each of the subpopulations [90]. In this case, K = 7 meets both criteria. To corroborate the STRUCTURE results, bootstrapped replicates (n = 1000) of collection locality pairwise Cavalli-Sforza chord distances were used to build a neighbor joining consensus tree. As visualized in Fig. 2, results from this analysis and STRUCTURE are congruent. The bootstrap results (values >50 shown) are similar to other studies that produced Cavalli-Sforza chord distance trees using species-specific microsatellites [91–93]. Differences among the detected subpopulations were further analysed by FCA analysis (Fig. 3). Axes 1 and 2 explain 25.34 and 21.37 % of the inertia, or variation, in individual *An. darlingi* genotypes. Two very distinct groups were detected, representing Central American (yellow) versus all South American collection localities (Fig. 3). Within the South American group, clear delineations can be seen between some of the subpopulations. As with the STRUCTURE and the Cavalli-Sforza chord distance tree (Fig. 2), *An. darlingi* from Peru collected in 2006 and 2012–2014 form two very distinct groups (fuchsia and green, respectively) with little overlap (Fig. 3).

**Fig. 4:**
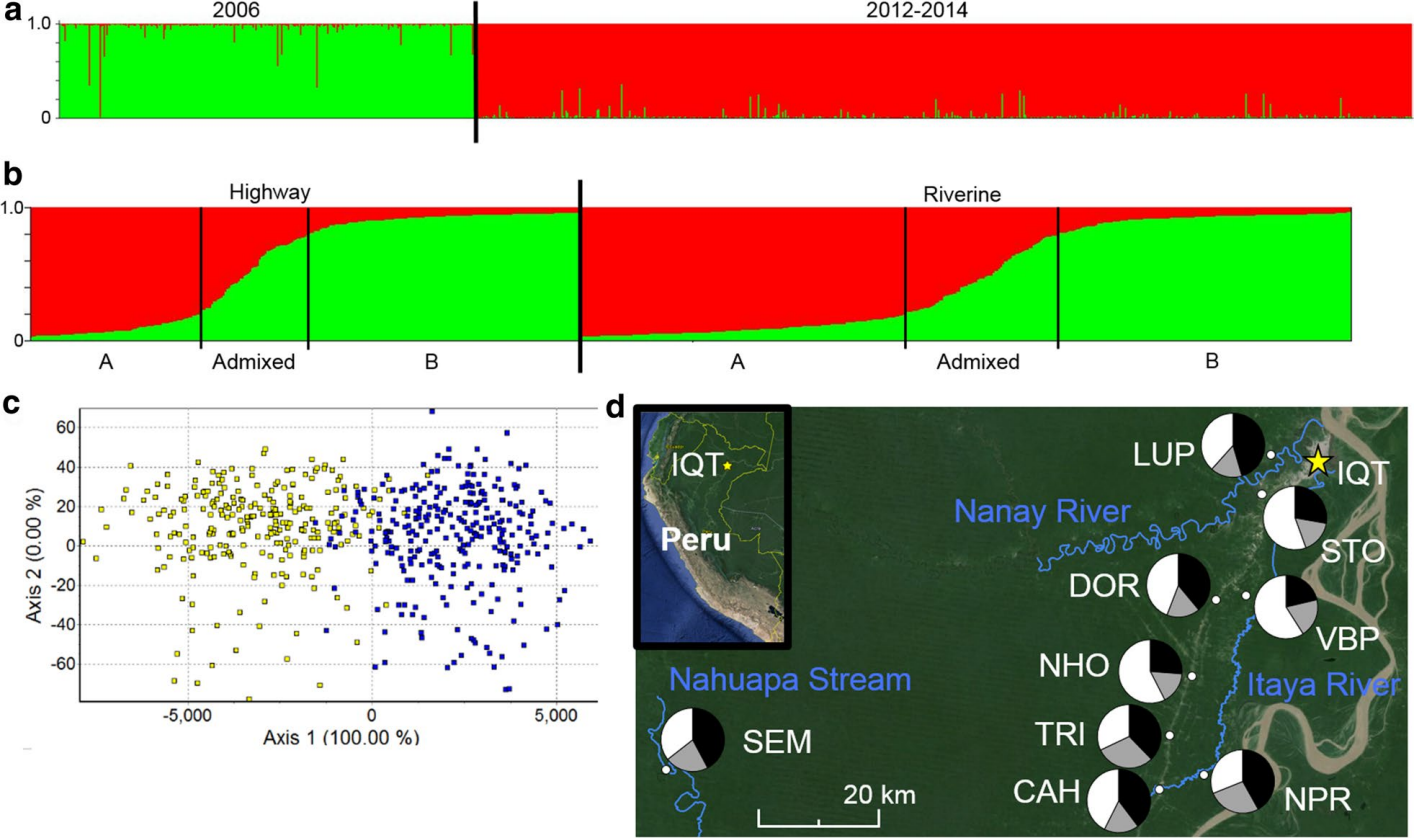
Population structure of *Anopheles darlingi*. **a** Results from the population structure analysis of mosquitoes from 2006 and 2012–2014 peri-Iquitos, Loreto, Peru collections, using 13 microsatellite loci. Optimal K = 2. **b** Results from the population structure analysis of mosquitoes collected in the current study, using 13 microsatellite loci. Optimal K = 2. Individuals within each habitat were sorted by subpopulation membership. *Vertical bars* within Highway and Riverine sections indicate cutoffs for membership to each subpopulation based on a threshold value of 0.8. Statistically significant differences in distribution of subpopulations by habitat ($\chi_{df = 2}^{2} = 1 1.0 6 4 8$, *p* < *0.005*). **c** Factorial Correspondence Analysis comparing subpopulations (13 microsatellite loci): *yellow* subpopulation *A*, *blue* subpopulation *B*. Axis *1* explains 100 % of the inertia in individual *An. darlingi* genotypes. **d** Map of the area surrounding Iquitos, Loreto, Peru (*yellow star*), depicting *An. darlingi* collection sites in this study (small *white circles*). The *pie charts* on the map, placed near the localities they represent, are the proportion of *An. darlingi* per detected subpopulation. Two subpopulations were detected in the current study (*black* subpopulation *A*, *white* subpopulation *B*, *grey* admixed). The Iquitos-Nauta highway can be seen in the satellite image, extending southwesterly from Iquitos. The Amazon river can be seen along the right-hand edge of the map

STRUCTURE analyses of the comparison between 2006 [46] and 2012–2014 (current study) *An. darlingi* resulted in an optimal K = 2, using 13 loci (Fig. 4a). The two groupings corresponded to the two collection periods, as predicted. One individual in the 2006 population is a member of the 2012–2014 cluster (Fig. 4a), as determined by STRUCTURE, indicating the presence of this cluster, albeit in a small proportion, in 2006. KS tests identified 9 of 13 loci (ADA03, ADA32, ADA40, ADC02, ADC110, ADC137, ADC28, ADMP9, ADSP2) with statistically significant differences in allele distributions between the 2006 and 2012–2014 *An. darlingi* populations (Table 1). Moderate differentiation was detected between the two populations (*F*_*ST*_ = 0.061).

**Table 1:** Testing the differences in allelic distributions per locus between 2006 and 2012–2014 peri-Iquitos, Loreto, Peru *Anopheles darlingi* populations

Locus	Kolmogorov–Smirnov one-sided test *p*-values
ADA03	*0.0029*
ADA20	0.3361
ADA32	*0.0129*
ADA40	*0.0293*
ADA41	0.7881
ADC02	<*2.2* × *10* ^−*16*^
ADC107n	0.1836
ADC110	*6.93* × *10* ^−*16*^
ADC137	*0.0128*
ADC138	0.6186
ADC28	<*2.2* × *10* ^−*16*^
ADMP9	*0.0198*
ADSP2	*0.0001*

Italics values indicate statistically significant one-sided Kolmogorov–Smirnov tests

STRUCTURE reanalyses of the 2006 peri-Iquitos *An darlingi* population using 13 loci, resulted in an optimal K = 2 (Additional file 5B). The population of *An. darlingi* within the five localities from the same region as the current study was composed of two highly admixed subpopulations; in contrast the *An. darlingi* population in the two localities to the east, near the Brazilian border, was composed of primarily one subpopulation, with little admixture (Additional file 5A, B).

The population genetics of *An. darlingi* from the current study (collected 2012–2014) were initially analysed using 14 microsatellite loci. However, because two of these loci (ADA27 and ADC137) were in linkage disequilibrium (*p* < 0.0001), locus ADA27 was removed and the data were re-analysed using the remaining 13. STRUCTURE and STRUCTURE Harvester results of *An. darlingi* show two highly admixed subpopulations (optimal K = 2): subpopulations A and B (Fig. 4b). A statistically significant difference in distribution of the subpopulations between habitats was detected ($\chi_{df = 2}^{2} = 1 1.0 6 4 8$, *p* < 0.005, Fig. 4b, d). Whereas subpopulations A and B are present in approximately equal proportions in the riverine collections (42.08 vs. 37.87 %, respectively), subpopulation B is overrepresented in highway collections compared with A (49.48 vs. 30.80 % respectively). FCA (Fig. 4c) depicts two distinct clusters of genotypes with little overlap in this study: subpopulation A (yellow) and B (blue); axis 1 explains 100 % of the inertia among individual *An. darlingi* genotypes. Differences in the distribution of subpopulations among forest cover levels were not significantly different ($\chi_{df = 4}^{2} = 3. 40 4 8$, *p* < 0.50).

### Measures of diversity within identified subpopulations

No significant differences were detected in *A*, *P*_*A*_, *H*_*E*_ or *H*_*O*_ between subpopulations A and B (Table 2). Evidence of expansion in both subpopulations was detected (Wilcoxon test, SMM model, one tail for H deficiency, *p* = 0.00018 and *p* = 0.00006, A and B, respectively). Consistent with population expansion, 22 of 26 exact tests for HWE were significant, and positive *F*_*IS*_ values were consistent with inbreeding in both subpopulations (Table 2). Genetic differentiation between subpopulation A and B was moderate (*F*_*ST*_ = 0.051), according to Hartl and Clark [94], with moderate gene flow (*N*_*M*_ = 4.65; Table 2). After removal of outlier locus ADC107n, as determined by LOSITAN, the two subpopulations showed little genetic differentiation (neutral *F*_*ST*_ = 0.021586) with moderate-high gene flow (neutral *N*_*M*_ = 11.33, Table 2).

**Table 2:** Measures of diversity, differentiation and gene flow for subpopulations identified in this study

Subpopulation	All loci^a^
Current study (A)	
*A*	10.0
*P*_*A*_	1.5
*H*_*E*_	0.711
*H*_*O*_	0.486
*F*_*IS*_	0.317
Current study (B)	
*A*	11.0
*P*_*A*_	2.7
*H*_*E*_	0.700
*H*_*O*_	0.458
*F*_*IS*_	0.345
Overall	
*F*_*ST*_	0.051
*N*_*M*_	4.65
Neutral	
*F*_*ST*_	0.022
*N*_*M*_	11.33

*A* number of alleles, *P*_*A*_ number of private alleles, *H*_*E*_ expected heterozygosity, *H*_*O*_ observed heterozygosity, *F*_*IS*_ inbreeding coefficient, *F*_*ST*_ population differentiation, *N*_*M*_ number of migrants (gene flow)

^a^Thirteen microsatellite loci

### Forest cover, collection, HBR and EIR

Across all forest cover levels, riverine settlements had significantly more forest cover than highway settlements (*p* = 0.0034, 7.4 × 10^−6^ and 0.0452 for SI50, SI100 and HP, respectively, Additional file 6). Additionally, both peridomestic (*p* = 5.9 × 10^−14^, 1.27 × 10^−11^ and <2 × 10^−16^, respectively) and *chacra* (*p* = 1.65 × 10^−6^, 9.89 × 10^−5^ and <2 × 10^−16^, respectively) had significantly less forest cover than forest collection sites, regardless of habitat. There was no significant effect of the interaction on forest cover level and habitat on calculated forest cover (*p* = 0.351, 0.16338 and 0.102, respectively), and no significant differences among forest cover calculation methods (adjusted R^2^: SI50 vs. SI100 = 0.8866, SI50 vs. HP = 0.8201, and SI100 vs. HP = 0.6983).

*Anopheles darlingi* (n = 2067) were collected between April and June 2014 in DOR, NHO, TRI, CAH and NPR. On average, the *An. darlingi* HBR was 17.64 bites per person per 12-h night. Of the mosquitoes collected, 0.63 % were infected (n = 13; *P. vivax*—nine, *P. falciparum*—three, *Plasmodium* spp.—one), resulting in an overall EIR of 0.11 infective bites per person per 12-h night (~3.5 per month; Table 3).

**Table 3:** The number of *Anopheles darlingi* collected (April–June 2013), HBR, IR and EIR

Habitat	Loc	Forest cover level	# Coll	# Coll (mh)	HBR	# Infected			IR (%)	EIR
						Pv	Pf	Pspp		
All	All	All	2067	1406	17.64	9	3	1	0.63	0.11
All	All	Peridomestic	1778	474	45.01	8		1	0.51	0.23
		Chacra	193	466	4.97	1	3		2.07	0.10
		Forest	96	466	2.47					
Riverine	All	All	1762	550	38.44	7	2		0.51	0.20
	All	Peridomestic	1552	186	100.13	6			0.39	0.39
		Chacra	137	182	9.03	1	2		2.19	0.20
		Forest	73	182	4.81					
Highway	All	All	305	856	4.28	2	1	1	1.31	0.06
	All	Peridomestic	226	288	9.42	2		1	1.33	0.13
		Chacra	56	284	2.37		1		1.79	0.04
		Forest	23	284	0.97					

Four, approximately-monthly, 12-h collections; Loc, locality; # Coll, number collected; mh, man-hours; HBR, human biting rate: the number of bites per person per 12-h night; IR, infection rate: the proportion of collected *An. darlingi* infected with *Plasmodium*; EIR, entomological inoculation rate: the number of infective bites per person per 12-h night; Pv, *Plasmodium vivax*; Pf, *Plasmodium falciparum*; Pspp, *Plasmodium* spp.

There was a significant effect of forest cover level on HBR (*p* = 2.15 × 10^−6^). *Anopheles darlingi* were collected most frequently in peridomestic collections (n = 1778) compared with *chacra* (n = 193) and forest (n = 96). HBR was 45.01 in peridomestic collections, and approximately 9-fold lower (4.97) and 18-fold lower (2.47) in *chacra* and forest collections, respectively. Infected mosquitoes were collected only in peridomestic (eight *Plasmodium vivax*, one *Plasmodium* spp.) and *chacra* (one *P. vivax* and three *P. falciparum*). Of the *An. darlingi* collected near homes, 0.51 % were infected, resulting in an EIR of 0.23 infective bites per person per night (~7 per month). However, in the *chacra*, 2.07 % of *An. darlingi* were infected, resulting in an EIR of 0.10 infective bites per person per night (~3 per month; Table 3). Thus the risk of *Plasmodium* transmission to humans is 2.3-fold higher in peridomestic *versus chacra*.

The effect of habitat on human biting rate (*p* = 7.23 × 10^−6^) was significant. Riverine collections had an overall HBR of 38.44 compared to 4.28 for highway collections. Nine infected *An. darlingi* were collected in riverine localities (seven *P. vivax* and two *P. falciparum*), resulting in an EIR of 0.20 infective bites per person per night (~six per month), and four in highway localities (two *P. vivax*, one *P. falciparum* and one *Plasmodium* spp.) resulting in an EIR of 0.06 infective bites per person per night (~1.8 per month; Table 3). Therefore, the risk of *Plasmodium* transmission to humans is 3.33-fold higher in riverine compared to highway localities.

Habitat and forest cover level had a significant interaction effect on HBR (*p* = 3.99 × 10^−11^). Peridomestic collections in riverine settlements had higher HBR (100.13 vs. 9.42) and EIR (0.39 vs. 0.13) compared to the same collections in highway settlements. The pattern was very similar for *chacra* collections (HBR: 9.03 vs. 2.37, and EIR: 0.20 vs. 0.04; Table 3). Risk of *Plasmodium* transmission to humans was three-fold higher in riverine versus highway peridomestic collections and five-fold higher in riverine versus highway *chacra* collections.

Calculated forest cover using SI50, SI100 and HP had no significant effect on HBR (*p* = 0.0904, 0.5679 and 0.0555), though the effect of forest cover as calculated by SI50 and HP approached significance. However, forest cover calculated using SI50 and HP had a significant effect on EIR (*p* = 0.01501 and 0.03130, respectively), whereas SI100 did not (*p* = 0.0745; Table 3, Additional file 6). To assess whether distance from human and domestic animals, and not forest cover, was statistically associated with HBR, the distance between the peridomestic, *chacra* and forest collection sites was measured using ArcGIS 10.2.2. These distances were used in three linear models (one for each forest cover calculation method), and adjusted for both calculated forest cover and habitat. In all three models, increasing distance from the peridomestic collection site resulted in a significant decrease in HBR (*p* = 0.00651, *p* = 0.00185 and *p* = 0.00342, models adjusting for S150, SI100 and HP, respectively).

### *Anopheles darlingi* biting time

**Fig. 5:**
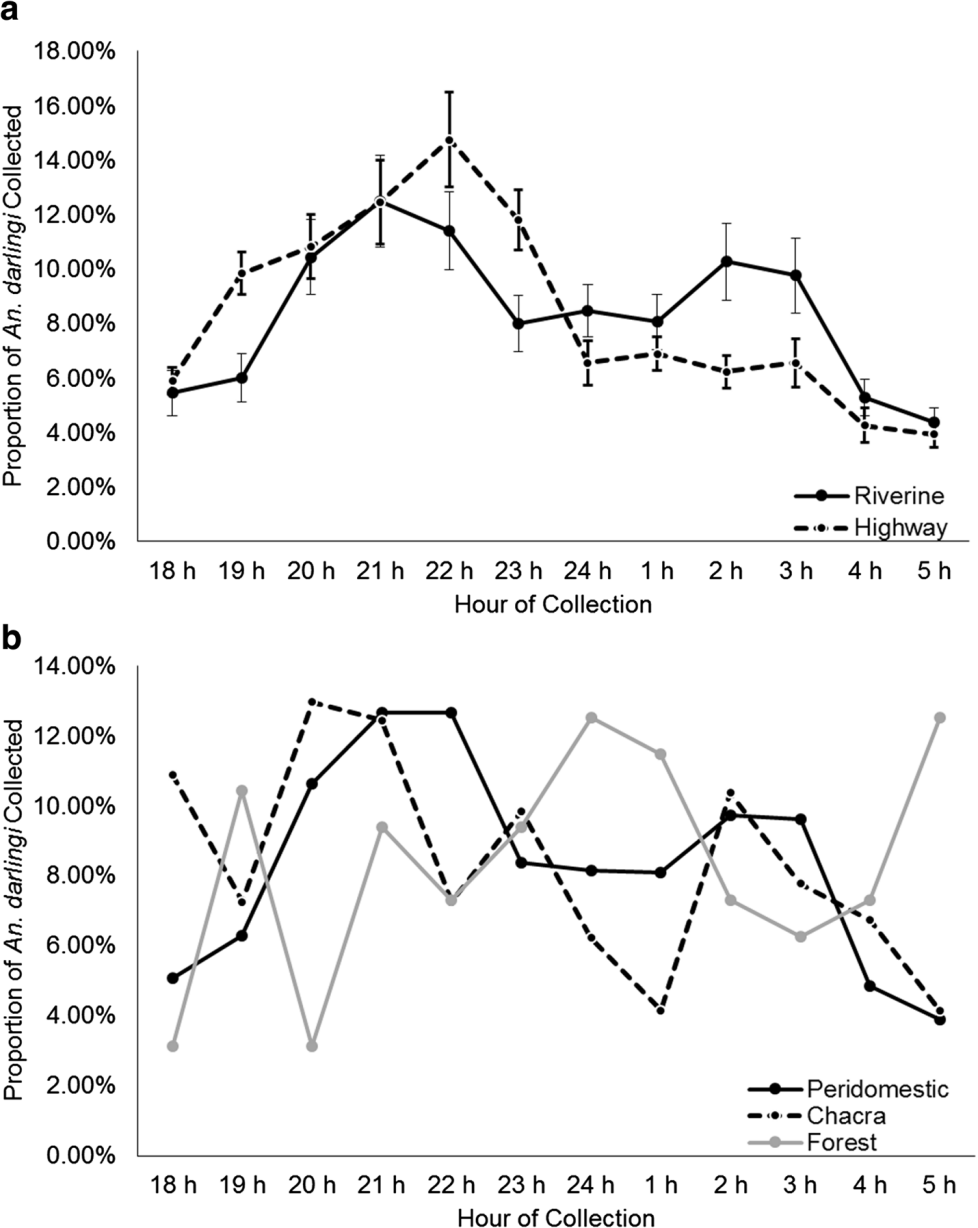
Average proportion of *An. darlingi* collected per hour in deforestation sites from April–June 2013. Data represent four, approximately monthly 12-h collections; SEM excluded. **a** Statistically significant differences noted by habitat: riverine settlements—*solid line*, highway settlements—*dashed line* (Kolmogorov–Smirnov test, one-sided, *p* = 0.001278). *Error bars* 95 % confidence intervals. **b** By forest cover level: peridomestic—*solid black line*, *chacra*—*dashed black line*, forest—*solid grey line* (Kolmogorov–Smirnov test, one-sided, peridomestic vs. *chacra* *p* = 0.05604, peridomestic vs. forest *p* = 0.02862, *chacra* vs. forest *p* = 0.01633). 95 % confidence intervals not shown

Significant differences in *An. darlingi* human biting were detected between riverine (bimodal peaks: 20:00–22:00, 01:00–03:00) and highway habitats (unimodal peak: 21:00–23:00), as tested by Kolmogorov–Smirnov test (one-sided, *p* = 0.001278; Fig. 5a). Overall, a significant difference in biting time pattern was detected between both peridomestic and forest (one-sided, *p* = 0.02862), and *chacra* and forest (one-sided, *p* = 0.01633), but not between peridomestic and *chacra* collections (one-sided, *p* = 0.05604; Fig. 5b). No significant differences were detected in biting times by subpopulation membership among all collected *An. darlingi* (*p* = 0.5248) or among *An. darlingi* collected for the deforestation study (*p* = 0.4566).

### *Anopheles darlingi* wing lengths

A total of 470 *An. darlingi* collected in deforestation sites were evaluated for differences in wing length by habitat, forest cover level and their interaction, and by subpopulation membership. A linear model showed no relationship between *An. darlingi* average wing length and habitat (*p* = 0.1991). However, the effect of forest cover level on average wing length was significant. Mosquitoes captured in forest and *chacra* sites did not differ in mean wing length (3.193 and 3.194 mm, respectively), whereas those in peridomestic sites had significantly shorter wings (3.121 mm, *p* = 0.0021). There was no significant interaction between habitat and forest cover (*p* = 0.6592), and no effect of subpopulation membership on average wing length (one tailed t-test, *p* = 0.4488).

## Discussion

Unexpectedly, a population replacement event in *An. darlingi* in Peru between 2006 and 2012–2014 was detected, using a Cavalli-Sforza chord distance tree (Fig. 2), Bayesian analyses with 13 microsatellite loci (Fig. 4a) and comparing allele frequency distributions per locus among the two populations (Table 1). The Cavalli-Sforza tree, despite its reliance on a fixed evolutionary rate, together with the STRUCTURE results (five microsatellite loci), suggest that the current Iquitos *An. darlingi* population is most closely related to mosquitoes from Amazonian Bolivia (1991, Fig. 2), but additional samples from southern Colombia and western Amazonas state in Brazil (Fig. 1), and more contemporary collections from the other regions need to be examined to discover the source of the current population. The significant population substructure within *An. darlingi* between Central and South America, as in previous studies [46], was confirmed. The results of the STRUCTURE analyses using only the 2006 *An. darlingi* collections, reanalysed with 13 loci, were different from those reported by Mirabello et al. using five loci [46] and Pinedo-Cancino et al. using AFLPs [50], which reported a single panmictic population of *An. darlingi* in the peri-Iquitos area. The reanalysed 2006 population was comprised of two subpopulations with different distributions. Among the localities within the region of the current study (MAZ, NAU, PCO, ZUN, PRT) the subpopulations were highly admixed, whereas *An. darlingi* collected in the two localities east of Iquitos (SPH: 175 km; SAE: 305 km) were primarily from one subpopulation, with less admixture (Additional file 5A, B).

Over a time period similar to that of the current study, a Suriname population of *An. darlingi* collapsed following the introduction of ITNs. Before the 2006 ITN distribution in Suriname, *An. darlingi* comprised 88.1 % of anophelines collected, with an average HBR of 1.43 bites per person per hour in three study sites. By 2009, post-ITN distribution, *An. darlingi* HBR dropped to 0 in the three sites [57]. However, the drop in HBR also coincided with abnormal flooding of rivers throughout the country [57], thus it was not possible to determine the effects of ITN due to the confounding effect of flooding, although decreases in *An. darlingi* density after inundation of larval habitats by higher than average rainfall and river levels have been documented previously in French Guiana and Brazil [95–97].

In 2007, under the programme PAMAFRO, the Organismo Andino de Salud (OAS, Andean Health Organization) began distributing LLINs throughout Colombia, Ecuador, Peru and Venezuela to reduce/eliminate regional *Plasmodium* transmission in concert with the push for global malaria eradication [98]. By 2010, when the campaign ended, OAS reported the distribution of approximately 346,000 LLINs protecting over 650,000 people. Between July and September 2007, 26,000 LLINs were provided to 194 targeted villages in Loreto, covering over 45,000 people. In a December survey of 60 villages, nearly 100 % of households owned an LLIN, with 99 % of children and 96 % of pregnant women reporting use the previous night [99]. A follow-up survey in July 2008 found that ownership had dropped only slightly (to 98.7 %) but usage by children and pregnant women had decreased to 77.7 and 66.3 %, respectively [99].

El Niño Southern Oscillation (ENSO) events from 2006–2014 have been primarily attributed to La Niña, which produces dry conditions with reduced precipitation in Peru, though there was a moderate-level El Niño event from July 2009–April 2010 [100], that resulted in peak river levels greater than 10 m in April 2011, and high monthly average river levels from August 2010–April 2011, compared to previous years [101]. Though it cannot be appropriately tested given the study design, possibly the PAMAFRO distribution of LLINs, together with the increased river levels at the end of the normally dry season in 2010 when *An. darlingi* numbers are typically low, contributed to the replacement event we identified. Disconcertingly, the number of malaria cases in Loreto has dramatically increased every year since 2011, despite decreases in the rest of Peru [36]. In 2010, there were 11,504 cases in Loreto (39.01 % of cases in Peru) and 60,186 cases (93.7 % of cases) in 2014 (see Additional file 7). The timing of this increase occurs just after both the LLIN distribution efforts and the 2009–2010 El Niño event, concomitantly with the population replacement event. The impact of this replacement event on the epidemiologies of *P. vivax* and *P. falciparum* appears to be minimal. Since 2008, the percentage of cases in Loreto due to *P. vivax* has hovered around 80 % (range 78.9–84.7 %, with the minimum and maximum occurring in 2011 and 2009, respectively) [36].

Within the current peri-Iquitos *An. darlingi* population, Bayesian analyses detected subpopulations with differential distribution between highway and riverine habitats, but not among forest cover levels, suggesting local adaptation to human altered habitat. Though population B is significantly overrepresented in highway settings, further study, such as larval site surveys, is needed to confirm this potential adaptation. Previous studies in the Peruvian Amazon have linked *An. darlingi* presence and malaria transmission with the density and size of fish ponds [55, 102]. Survey of these ponds for an overrepresentation of population B larvae would provide evidence for the adaptation hypothesis. Recently, in western Amazonian Brazil, two subpopulations of *An. darlingi* were detected using microsatellites, whose distributions were associated with seasonal differences: “typical” *An. darlingi* most abundant in the late rainfall period, and an anthropogenically-adapted group most abundant in the early rainfall period [56]. This second group is thought to use permanent, human-made larval sites since it is able to reach greater population size in the early rainfall season, before natural larval habitats are created, and persists in lower numbers than the first group throughout the remainder of the rainy season [56].

Vittor et al. [25, 55] conducted studies on the effect of forest cover and deforestation on biting rates and distribution of *An. darlingi* in peri-Iquitos. These studies detected a significant association between highly deforested sites within villages and increased HBR in *An. darlingi*, and were restricted to collection sites within highway habitat, to prevent confounding effects of riverine ecology on *An. darlingi* biting frequency. Within these sites, *An. darlingi* was consistently collected using human landing catch unimodally before midnight. The results in highway habitat collections of the current study were very similar, i.e., a significant effect of forest cover level on HBR, and unimodal biting before midnight. The 6-h HBRs (1800–24:00 h) calculated using the current study data (5.91, 1.79 and 0.63 in peridomestic, *chacra* and forest collections, respectively) were comparable to those of Vittor et al. [25] at equivalent forest cover levels 6.5 (4.9–8.0), 1.7 (1.0–2.4) and 0.0 (0.0–0.1) with increasing forest cover percentage.

However, the HBR was also significantly affected by habitat and the interaction between habitat and forest cover level. HBRs in riverine localities at the three forest cover levels were higher than their counterparts in highway localities, with the highest in peridomestic riverine collections. Additionally, *An. darlingi* biting in riverine collections was bimodal and statistically different from that of *An. darlingi* in highway. Peridomestic collection localities CAH and NPR (both riverine) had the highest HBRs, though they differed greatly in calculated forest cover percentage (Additional file 6). This result suggests that variation in HBR cannot be explained by forest cover percentage, but is likely a function of mosquito density (increased larval habitat in riverine habitat vs. highway), and increased biomass, including humans and domesticated animals [103]. To test this, distance from the peridomestic collection site (to the *chacra* and forest sites) was used as a proxy measure of biomass (increasing distance with decreasing biomass). After accounting for calculated forest cover and habitat, HBR was statistically significantly associated with distance, where HBR decreased with increasing distance from the peridomestic site (*p* = 0.000342, 0.000651 and 0.000185, for HP, SI50 and SI100, respectively). Similar to the findings of Navia-Gine et al. [103], *An. darlingi* biting is proportional to the available host biomass in a given area.

Differences were also detected among forest cover levels with respect to biting pattern. Overall, peridomestic and *chacra* collections exhibited bimodal biting before and after midnight, with unimodal biting after midnight in forest collections. These patterns may be a function of the proximity of the collection sites to human populations and/or breeding sites [104, 105], a function of host choice [105], and/or a function of host availability [106]. Of the three forest cover level biting patterns, the only pattern with peaks at both dawn and dusk is that of the forest (Fig. 5b). This suggests that *An. darlingi* that were more zoophilic were collected from forest habitat which, according to Charlwood’s hypothesis [105], would exhibit crepuscular biting, as opposed to more anthropophilic *An. darlingi*, which would bite nocturnally.

Overall, a *Plasmodium* infection rate (IR) of 0.63 % was detected among *An. darlingi* collected during the peak malaria transmission period (April–June) in 2013 (Table 3). Despite the 2.5-fold higher IR in highway versus riverine collections (1.31 and 0.51 %, respectively), people living in riverine communities are at a threefold greater risk of *Plasmodium* infection due to the much greater HBR in this habitat. This pattern of risk was also detected in peridomestic versus *chacra* collections (IR: 0.51 and 2.07 %, and EIR: 0.23 and 0.10, respectively), and in the interaction between habitat and forest cover level (threefold greater risk in riverine vs. highway peridomestic collection sites, and fivefold greater risk in riverine vs. highway *chacra* collection sites). In this study, no *Plasmodium* transmission attributed to *An. darlingi* was detected in the forest, since *An. darlingi* are rarely captured in forest collections, even those only 300 m from a peridomestic collection site [25, 31, 107, 108]. The overall *Plasmodium*, *P. vivax* and *P. falciparum* IRs (0.63, 0.44 and 0.15 %) determined in this study are similar to those published for *An. darlingi* collected in the peri-Iquitos region in 2008 and 2009 [34]. Whereas the *An. darlingi* IRs differed by season of collection in the Parker et al. [34], the IRs of the current study agree with those from specimens in [34] collected at the same time of year (April 2008; IRs = 0.89, 0.74, and 0.15 %, for *Plasmodium*, *P. vivax*, and *P. falciparum*, respectively). The IRs in the current study concur with previously reported rates in Brazil [109, 110]. Assuming PAMAFRO LLIN distribution and the 2010 ENSO event played a role in the population replacement event, the comparison of IRs between those reported by Parker et al. [34] and the current study demonstrate that the replacement event did not affect *An. darlingi* vector competence in the peri-Iquitos area.

Among the deforestation study samples that were genotyped using microsatellites (n = 156 and 173 for subpopulation A and B, respectively), eight were found to be infected with *Plasmodium*. Overall, subpopulation B had a higher *Plasmodium* IR (2.31 vs. 1.28 %) compared with A. *Plasmodium*-specific IRs in subpopulation B were also nearly double those of subpopulation A for both *Plasmodium* species (1.16 % for both *P. vivax* and *P. falciparum* in B versus 0.64 % for both *P. vivax* and *P. falciparum* in A). This finding has implications for malaria epidemiology in the peri-Iquitos region, especially if the hypothesis of subpopulation B adaptation to human-altered environments is correct, allowing it to transmit year round.

A significant effect of forest cover level on wing length in *An. darlingi* suggests that larval habitats differ in quality among the three forest cover levels, since wing length is highly correlated to adult body size. Mosquito larvae living in nutrient-rich habitats become larger adult mosquitoes, whereas nutrient-poor habitats produce smaller adults [111]. A similar effect occurs as a result of increased competition in larval habitats in *An. gambiae* [112, 113]. An additional, non-mutually exclusive, explanation is changes in microclimatic conditions due to local deforestation [114]. In Kenya, deforested sites have been shown to experience higher midday temperatures than forested sites [115, 116]. In *An. gambiae*, adult body size was negatively correlated with larval rearing temperature [117]. Additionally, temperature has been shown to have differing effects on mosquito development at different life stages and ultimately influences mosquito population dynamics and adult population age structure, with implications for vectorial capacity [118]. Body size has been shown to be positively correlated with vectorial capacity among mosquito vectors, due to the availability of fat stores and the energy needed to maintain *Plasmodium* infection [111], whereas temperature has a more complex relationship with *Plasmodium*, with peak transmission occurring at 25 °C [119]. Decreased larval nutrition in *An. darlingi* is associated with increased biting frequency because these undernourished mosquitoes required more than one blood meal to complete a gonotrophic cycle [120], though the opposite result was seen in a separate study [121]. The decreased average wing length, and therefore body size, and presumed increased local temperature in peridomestic sites, may explain the decreased *Plasmodium* IR among these mosquitoes, compared to those from the *chacra* sites (overall 0.51 and 2.07 %, respectively; Table 3). However, the increased number of *An. darlingi* collected in peridomestic sites overcomes this difference, ultimately resulting in a twofold increased risk of malaria compared to *chacra* (Table 3).

## Conclusions

Taken together, these results show that *An. darlingi* in Loreto, Peru underwent a drastic population replacement event after 2006 [46], consistent with the temporal population replacement hypothesis, though the role of the PAMAFRO LLIN distributions is confounded by a concurrent El Niño event. Population replacement of vectors is of great interest because new populations may be less susceptible to interventions currently in use, and/or may have altered behaviours and vectorial capacity. Substructuring within the new population collected between 2012 and 2014 was detected. These subpopulations differ in their distributions among highway and riverine habitats and *Plasmodium* IR, but not wing length, and may be in the early stages of adaptive divergence, providing evidence of ecological adaptation to human altered environments. Whereas HBR and biting pattern do not differ by subpopulation, these important vector biology metrics were associated with habitat and forest cover level, consistent with habitat differentiation. The results of this study show the need for a focus on vector control intervention in riverine settlements, which are hot spots of malaria transmission and risk in this region.

## Additional files

10.1186/s12936-015-0863-4 *Anopheles darlingi* collection information.
10.1186/s12936-015-0863-4 Exemplar images depicting forest cover determination using satellite imagery. For each of the three forest cover levels, forest cover was calculated at both 50 m (inner circle) and 100 m (outer circle) radii from the collection site. Non-forest areas of the image were selected and colored pure black. The percentage of non-black pixels was determined using GIMP v.2.8.10 image software [64].
10.1186/s12936-015-0863-4 Exemplar images depicting forest cover determination using hemispherical photography. For peridomestic and *chacra *forest cover levels, 30 randomly selected sites within 100 m of the collection site were chosen. At each site, a hemispherical image was taken directly upward to quantify the canopy coverage. Only one image was taken at the forest site, due to safety concerns. Canopy coverage in each image was determined using CAN-EYE v.6.314 [67] hemispherical image analysis software. In the lower images, a building was masked in the lower left corner, to prevent its classification as forest cover.
10.1186/s12936-015-0863-4 *Anopheles darlingi*-specific microsatellite loci employed in this study and the length of the 72°C extension step at the end of the PCR program for each.
10.1186/s12936-015-0863-4 Reanalysis of *Anopheles darlingi* collected in the peri-Iquitos region in 2006, using microsatellites. (**A**) *Anopheles darlingi* collection sites from 2006 (white squares) and 2012-2014 (grey circles) near Iquitos, Loreto, Peru (yellow star). (**B**) Population structure of *Anopheles darlingi* collected in the peri-Iquitos region in 2006, reanalyzed using thirteen microsatellite loci. Collection of these specimens was previously described by Mirabello*et al.* [46]. Optimal K = 2. MAZ = Mazan; NAU = Nauta; PCO = Padre Cocha; ZUN = Zungarococha; PRT = Piura, Rio Tigre; SHP = Shishita, Pevas; SAE = San Esteban.
10.1186/s12936-015-0863-4 Forest cover percentage determined using satellite imagery and hemispherical photography by locality and forest cover level.
10.1186/s12936-015-0863-4 Number of malaria cases (*Plasmodium vivax *and *Plasmodium falciparum*) in all of Peru (solid line, triangle markers) versus Loreto department (dashed line, square markers) [36]. Timing of PAMAFRO long lasting insecticidal net distribution (LLIN; red solid line) and El Niño event (blue solid line) shown [98, 100].
