# Supplementary material for: Evidence for temporal population replacement and the signature of ecological adaptation in a major Neotropical malaria vector in Amazonian Peru
Source: Malar J. 2015 Sep 29;14:375. doi: 10.1186/s12936-015-0863-4 (PMC4587789; doi:10.1186/s12936-015-0863-4)
Supplement: Supplementary file 1 — 10.1186/s12936-015-0863-4 Anopheles darlingi collection information. [file 12936_2015_863_MOESM1_ESM.pdf]

Additional file 1. *Anopheles darlingi* collection information.

Locality	Forest Cover Level	Latitude	Longitude	# *An. darlingi* *	Distance from River	Dates of Collection
Current Study						
Cahuide (CAH)				141 (141)	0 km	March/October 2012, April - June 2013, May 2014
	Peridomestic	04° 13' 49.26" S	73° 29' 16.20" W	106		
	Chacra	04° 14' 55.21" S	73° 29' 40.92" W	14		
	Forest	04° 14' 26.88" S	73° 29' 55.00" W	21		
El Dorado (DOR)				77 (76)	5.91 km	April - June 2013, June 2014
	Peridomestic	03° 57' 18.60" S	73° 24' 18.48" W	26		
	Chacra	03° 57' 44.03" S	73° 23' 56.04" W	26		
	Forest	03° 58' 09.53" S	73° 23' 49.27" W	25		
El Triunfo (TRI)				53 (52)	8 km	April - June 2013, May 2014
	Peridomestic	04° 08' 58.32" S	73° 28' 25.02" W	17		
	Chacra	04° 09' 51.95" S	73° 28' 27.01" W	14		
	Forest	04° 09' 11.05" S	73° 28' 56.17" W	22		
Nuevo Horizonte (NHO)				80 (80)	6.68 km	April - June 2013, June 2014
	Peridomestic	04° 03' 54.30" S	73° 26' 28.62" W	27		
	Chacra	04° 04' 33.78" S	73° 26' 45.89" W	26		
	Forest	04° 04' 29.75" S	73° 26' 32.86" W	27		
Nuevo Progreso (NPR)				74 (71)	0.27 km (dry season), 0 km (wet season)	April - June 2013, May 2014
	Peridomestic	04° 12' 30.12" S	73° 25' 26.82" W	23		
	Chacra	04° 12' 34.20" S	73° 25' 35.58" W	27		
	Forest	04° 12' 56.59" S	73° 25' 26.62" W	24		
San Jose de Lupuna (LUP)	Peridomestic	03° 44' 35.45" S	73° 19' 36.91" W	99 (99)	0.84 km (dry season), 0 km (wet season)	February/April 2012
Santa Emilia (SEM)				90 (90)	0 km	October 2013 - February 2014, April - May 2014
	Peridomestic	04° 11' 58.99" S	74° 12' 20.12" W	30		
	Chacra	04° 11' 58.99" S	74° 12' 20.12" W	30		
	Forest	04° 11' 56.08" S	74° 12' 54.94" W	30		
Santo Tomas (STO)	Peridomestic	03° 48' 07.74" S	73° 20' 19.50" W	18 (18)	0 km	March/October 2012
Villa del Buen Pastor (VBP)	Peridomestic	03° 56' 59.82" S	73° 21' 39.24" W	61 (59)	2.32 km	February/October 2012
Mirabello *et al*. (2008)						
Belize						
Caves Branch (CAV)	n/a	17° 09' 00.00" N	88° 40' 01.80" W	34	n/a	March 2006
Golden Stream (GOL)	n/a	16° 21' 48.84" N	88° 47' 55.20" W	39	n/a	March 2006
Sibun (SIB)	n/a	17° 08' 53.22" N	88° 37' 41.34" W	24	n/a	March 2006
Guatemala						
El Penon (ELP)	n/a	16° 01' 47.64" N	90° 46' 29.64" W	28	n/a	September 2000
San Pablo (SPB)	n/a	15° 58' 05.88" N	90° 47' 24.00" W	31	n/a	September 2000
Santa Rosa (SRO)	n/a	15° 58' 38.64" N	90° 50' 53.88" W	39	n/a	September 2000
Brazil						
Boa Vista (BV)	n/a	02° 49' 00.00" N	60° 40' 00.00" W	56	n/a	July 2003
Palito (IT)	n/a	06° 33' 53.00" S	56° 31' 37.00" W	52	n/a	July 2003
Peru						
Mazan (MAZ)	n/a	03° 29' 32.32" S	73° 14' 30.52" W	48	n/a	February 2006
Nauta (NAU)	n/a	04° 30' 41.65" S	73° 35' 08.74" W	51	n/a	February 2006
Padre Cocha (PCO)	n/a	03° 42' 12.38" S	73° 16' 58.48" W	47	n/a	January 2006
Piura, Rio Tigre (PRT)	n/a	04° 06' 35.76" S	74° 25' 05.16" W	17	n/a	February/March 2006
Shishita, Pevas (SHP)	n/a	03° 22' 34.61" S	71° 43' 38.42" W	54	n/a	March 2006
San Esteban (SAE)	n/a	03° 56' 48.55" S	70° 30' 57.56" W	44	n/a	March 2006
Zungarococha (ZUN)	n/a	03° 49' 33.92" S	73° 21' 04.72" W	50	n/a	January 2006
Conn *et al*. (2006)						
Brazil						
Aracanga (ARA)	n/a	01° 37' 00.00" S	48° 36' 00.00" W	38	n/a	1997-1999
Belem (BEL)	n/a	01° 41' 00.00" S	48° 40' 00.00" W	41	n/a	1997-2000
Moju (MOJ)	n/a	01° 52' 00.00" S	48° 45' 00.00" W	26	n/a	1997-2001
Peixoto de Azevedo (PEX)	n/a	10° 23' 00.00" S	54° 54' 00.00" W	23	n/a	1997-2002
Lagoa dos Indios (LI)	n/a	00° 02' 00.00" S	51° 11' 00.00" W	39	n/a	1997-2003
Granja Alves (GA)	n/a	00° 02' 00.00" S	51° 05' 00.00" W	40	n/a	1997-2004
Santana (STN)	n/a	00° 01' 00.00" S	51° 09' 00.00" W	39	n/a	1997-2005
Scarpassa and Conn (2007)						
Brazil						
Bancrevea (BAN)	n/a	02° 59' 00.00" S	60° 04' 00.00" W	32	n/a	April 1998
Ramal do Brasileirinho (RBR)	n/a	03° 01' 00.00" S	59° 57' 00.00" W	44	n/a	July 1998
Coari (COA)	n/a	04° 05' 00.00" S	63° 08' 00.00" W	43	n/a	June 2001
Castanho (CAS)	n/a	03° 49' 00.00" S	60° 21' 00.00" W	40	n/a	June 1998
Novo Airao (NAI)	n/a	01° 56' 00.00" S	61° 22' 00.00" W	34	n/a	December 1998
Porto Velho (PVE)	n/a	08° 45' 00.00" S	63° 53' 00.00" W	40	n/a	April 2002
Municipality of Acrelandia (MAC)	n/a	10° 08' 00.00" S	67° 32' 00.00" W	22	n/a	August 2002
Sao Miguel (SMI)	n/a	08° 36' 00.00" S	63° 49' 00.00" W	40	n/a	April 2002
Gutierrez *et al*. (2010)						
Colombia						
Montelibano (MTL)	n/a	07° 58' 48.00" N	75° 25' 12.00" W	25	n/a	2006-2008
Puerto Libertador (PLT)	n/a	07° 54' 00.00" N	75° 40' 12.00" W	38	n/a	2006-2008
El Bagre (BAG)	n/a	07° 34' 48.00" N	74° 48' 00.00" W	35	n/a	2006-2008
Zaragoza (ZAR)	n/a	07° 28' 48.00" N	74° 52' 12.00" W	7	n/a	2006-2008
Angêlla *et al*. (2014)						
Brazil						
Vila Candelaria (CAN)	n/a	08° 46' 42.20" S	63° 54' 59.50" W	16	n/a	2007-2008
Engenho Velho (ENV)	n/a	08° 46' 23.60" S	63° 55' 19.70" W	24	n/a	2007-2008
Jaci Parana (JAP)	n/a	09° 14' 30.00" S	64° 23' 44.00" W	21	n/a	2007-2008
Santo Antonio (STA)	n/a	08° 47' 25.40" S	63° 55' 25.70" W	10	n/a	2007-2008
Amazonas (AMA)	n/a	08° 50' 49.80" S	64° 02' 09.60" W	21	n/a	2007-2008
Teotonio (TEO)	n/a	08° 50' 20.50" S	64° 02' 18.00" W	15	n/a	2007-2008
Bate Estaca (BAT)	n/a	08° 46' 04.50" S	63° 54' 11.10" W	15	n/a	2007-2008
Moreno, unpubl.						
Bolivia						
Guayaramerin (BGU)	n/a	10° 49' 01.20" S	65° 24' 00.00" W	12	n/a	1991
Venezuela						
Fortuna de Albarico (FOR)	n/a	05° 58' 01.20" N	67° 25' 01.20" W	8	n/a	1992-1993

* Number of *An. darlingi* used in microsatellite analyses; Current study mosquitoes: number analyzed with 13 microsatellite loci (number analyzed with 5 microsatellite loci)
