# Supplementary material for: Evidence for temporal population replacement and the signature of ecological adaptation in a major Neotropical malaria vector in Amazonian Peru
Source: Malar J. 2015 Sep 29;14:375. doi: 10.1186/s12936-015-0863-4 (PMC4587789; doi:10.1186/s12936-015-0863-4)
Supplement: Supplementary file 2 — 10.1186/s12936-015-0863-4 Exemplar images depicting forest cover determination using satellite imagery. For each of the three forest cover levels, forest cover was calculated at both 50 m (inner circle) and 100 m (outer circle) radii from the collection site. Non-forest areas of the image were selected and colored pure black. The percentage of non-black pixels was determined using GIMP v.2.8.10 image software [64]. [file 12936_2015_863_MOESM2_ESM.pdf]

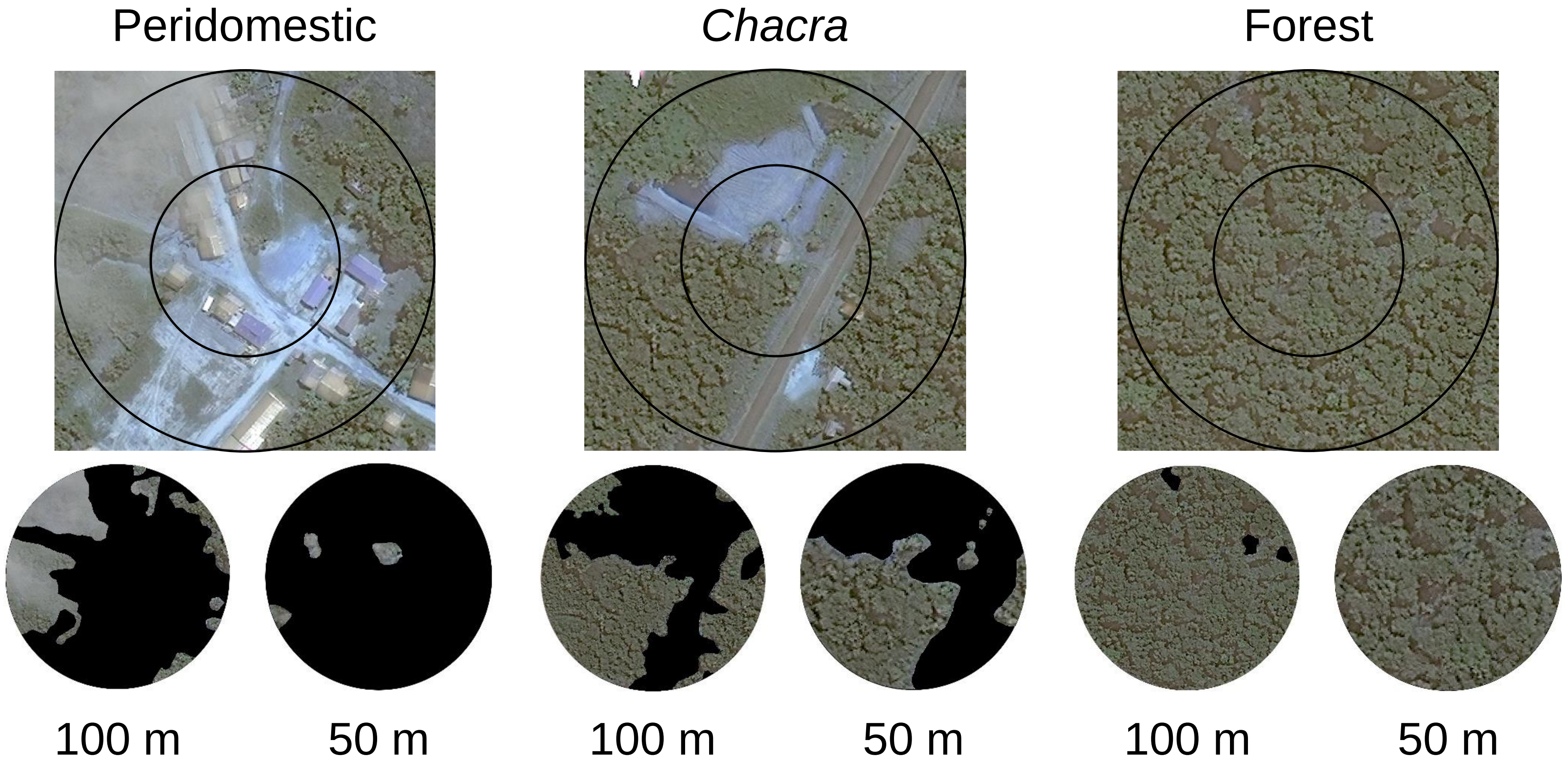

Additional file 2. Exemplar images depicting forest cover determination using satellite imagery. For each of the three forest cover levels, forest cover was calculated at both 50 m (inner circle) and 100 m (outer circle) radii from the collection site. Non-forest areas of the image were selected and colored pure black. The percentage of non-black pixels was determined using GIMP v.2.8.10 image software (www.gimp.org).
