# Supplementary material for: Evidence for temporal population replacement and the signature of ecological adaptation in a major Neotropical malaria vector in Amazonian Peru
Source: Malar J. 2015 Sep 29;14:375. doi: 10.1186/s12936-015-0863-4 (PMC4587789; doi:10.1186/s12936-015-0863-4)
Supplement: Supplementary file 3 — 10.1186/s12936-015-0863-4 Exemplar images depicting forest cover determination using hemispherical photography. For peridomestic and chacra forest cover levels, 30 randomly selected sites within 100 m of the collection site were chosen. At each site, a hemispherical image was taken directly upward to quantify the canopy coverage. Only one image was taken at the forest site, due to safety concerns. Canopy coverage in each image was determined using CAN-EYE v.6.314 [67] hemispherical image analysis software. In the lower images, a building was masked in the lower left corner, to prevent its classification as forest cover. [file 12936_2015_863_MOESM3_ESM.pdf]

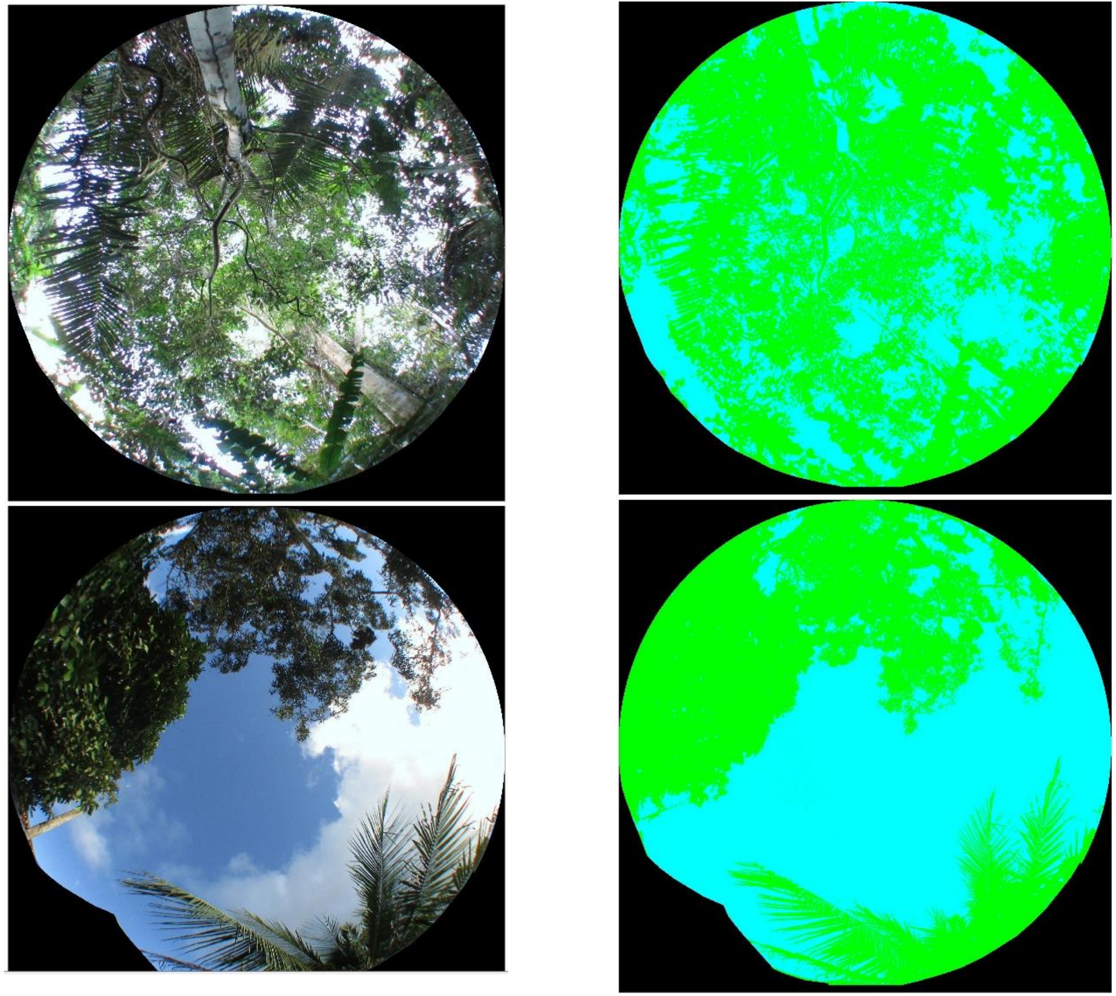

Additional file 3. Exemplar images depicting forest cover determination using hemispherical photography. For peridomestic and *chacra* forest cover levels, 30 randomly selected sites within 100 m of the collection site were chosen. At each site, a hemispherical image was taken directly upward to quantify the canopy coverage. Only one image was taken at the forest site, due to safety concerns. Canopy coverage in each image was determined using CAN-EYE v.6.314 (http://www6.paca.inra.fr/can-eye) hemispherical image analysis software. In the lower images, a building was masked in the lower left corner, to prevent its classification as forest cover.
