# Supplementary material for: Evidence for temporal population replacement and the signature of ecological adaptation in a major Neotropical malaria vector in Amazonian Peru
Source: Malar J. 2015 Sep 29;14:375. doi: 10.1186/s12936-015-0863-4 (PMC4587789; doi:10.1186/s12936-015-0863-4)
Supplement: Supplementary file 4 — 10.1186/s12936-015-0863-4 Anopheles darlingi-specific microsatellite loci employed in this study and the length of the 72°C extension step at the end of the PCR program for each. [file 12936_2015_863_MOESM4_ESM.pdf]

Additional file 4. Anopheles darlingi-specific microsatellite loci employed in this study and the length of the 72°C extension step at the end of the PCR program for each.

Locus	Forward/ Reverse	Primer Sequence (5' - 3')	Fluorophore	Length of Extension Step	Source
ADC02	F	CAC ACT GGG GCA TCA TTC ATT TC	FAM	5 minutes	Conn *et al*. (2001)
	R	TCA CAC TGC GTC CTT AGA CAC TG			
ADC28	F	CTC GTC GTC AGC GTC GTG C	FAM	5 minutes	Conn *et al*. (2001)
	R	TGC CCA TCC ACT GCG TAA CGG			
ADC110	F	CCG AAC AAC AGC CAA CAG CTG TG	HEX	5 minutes	Conn *et al*. (2001)
	R	CGT TCG ACA CAA TCG TTA CAC ACG			
ADC137	F	TCT TAC GGG AAT GGT GCG ACG CTC	HEX	5 minutes	Conn *et al*. (2001)
	R	CAG CCA CCC ATA CGC TGT TGA CCA			
ADC138	F	CTT TGA GCC GGT GCT GTG CTG C	FAM	30 minutes	Conn *et al*. (2001)
	R	CCA TTC TCG CAG CCT CCA GGA C			
ADA03	F	AGA GAG CTA ATG CGG TTG GTC	FAM	30 minutes	Lima *et al*. (2010)
	R	ACG TTC CTC TAC TCC GAA AGC			
ADA20	F	AGC AAT ATG TTC CCG ACA GC	FAM	30 minutes	Lima *et al*. (2010)
	R	CGG CTT CTA AAT GAC TCC TAG C			
ADA27	F	AGC GGA TCT ACC TAC GGG TTA	FAM	5 minutes	Lima *et al*. (2010)
	R	CGC TAT CAG CAT CAT CAT CG			
ADA32	F	TCA CTA GCG TAT GTG CGA GG	HEX	5 minutes	Lima *et al*. (2010)
	R	TCG AAT GAC CTT TGG GAG AC			
ADA39	F	GAT CGC AGT AGC TGA AAG TCG	HEX	5 minutes	Lima *et al*. (2010)
	R	GAA TAT CGC GGT GGA TCA G			
ADA40	F	TAC TAC TGA TTG GCG CTC CTG	FAM	30 minutes	Lima *et al*. (2010)
	R	ACT ACG GGT CCT CTC GTG TTC			
ADA41	F	CGC TGA GAA CAT TGG GTA GTC	FAM	5 minutes	Lima *et al*. (2010)
	R	GTG GTA CTG CGA GGA TCA AAG			
ADC107n	F	GTC CAC TCC CAG GCA CAC	HEX	30 minutes	Angêlla *et al*. (2014)
	R	AGC AAT CGA GGC AAA CTT TC			
ADSP2	F	GCA TAT ATT CTC GCC GCA TT	FAM	5 minutes	Angêlla *et al*. (2014)
	R	TCA GCT ACT ACC CGA CGA CA			
ADMP9	F	ACA ACG TCC AAT GCA ACA AC	HEX	5 minutes	Angêlla *et al*. (2014)
	R	CTC GAG GGC TTT CTG TAT CG			
