# Supplementary material for: Evidence for temporal population replacement and the signature of ecological adaptation in a major Neotropical malaria vector in Amazonian Peru
Source: Malar J. 2015 Sep 29;14:375. doi: 10.1186/s12936-015-0863-4 (PMC4587789; doi:10.1186/s12936-015-0863-4)
Supplement: Supplementary file 5 — 10.1186/s12936-015-0863-4 Reanalysis of Anopheles darlingi collected in the peri-Iquitos region in 2006, using microsatellites. (A) Anopheles darlingi collection sites from 2006 (white squares) and 2012-2014 (grey circles) near Iquitos, Loreto, Peru (yellow star). (B) Population structure of Anopheles darlingi collected in the peri-Iquitos region in 2006, reanalyzed using thirteen microsatellite loci. Collection of these specimens was previously described by Mirabelloet al. [46]. Optimal K = 2. MAZ = Mazan; NAU = Nauta; PCO = Padre Cocha; ZUN = Zungarococha; PRT = Piura, Rio Tigre; SHP = Shishita, Pevas; SAE = San Esteban. [file 12936_2015_863_MOESM5_ESM.pdf]

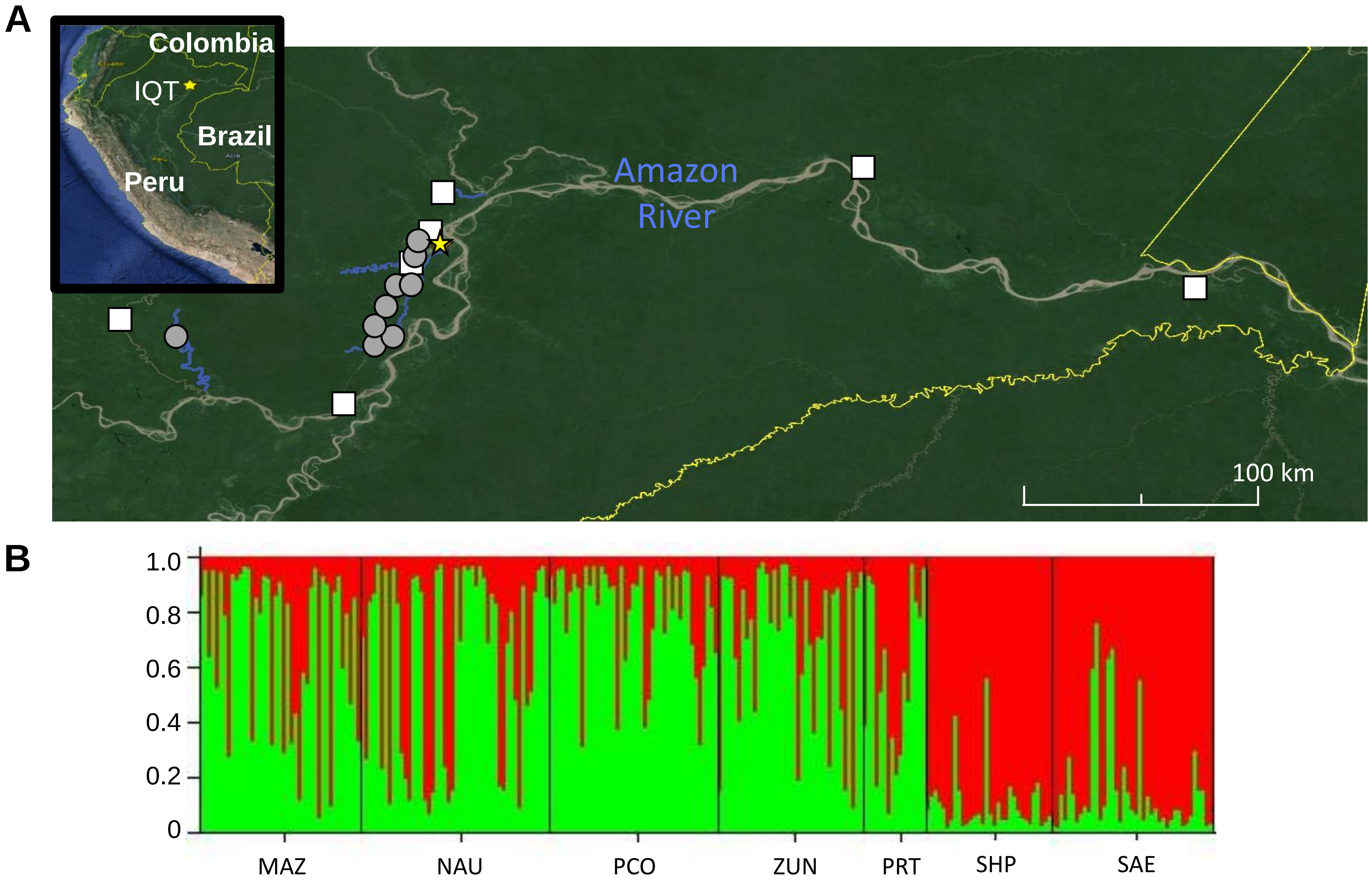

Additional file 5. Reanalysis of *Anopheles darlingi* collected in the peri-Iquitos region in 2006, using microsatellites. (**A**) *Anopheles darlingi* collection sites from 2006 (white squares) and 2012-2014 (grey circles) near Iquitos, Loreto, Peru (yellow star). (**B**) Population structure of *Anopheles darlingi* collected in the peri-Iquitos region in 2006, reanalyzed using thirteen microsatellite loci. Collection of these specimens was previously described by Mirabello *et al.* [46]. Optimal K = 2. MAZ = Mazan; NAU = Nauta; PCO = Padre Cocha; ZUN = Zungarococha; PRT = Piura, Rio Tigre; SHP = Shishita, Pevas; SAE = San Esteban.
