# Supplementary material for: Evidence for temporal population replacement and the signature of ecological adaptation in a major Neotropical malaria vector in Amazonian Peru
Source: Malar J. 2015 Sep 29;14:375. doi: 10.1186/s12936-015-0863-4 (PMC4587789; doi:10.1186/s12936-015-0863-4)
Supplement: Supplementary file 6 — 10.1186/s12936-015-0863-4 Forest cover percentage determined using satellite imagery and hemispherical photography by locality and forest cover level. [file 12936_2015_863_MOESM6_ESM.pdf]

**Additional file 6**. Forest cover percentage determined using satellite imagery and hemispherical photography by locality and forest cover level.

| **Habitat** | **Locality** | **Forest Cover Level** | **Calculated Forest Cover** | | |
|---|---|---|---|---|---|
| | | | **Satellite Imagery (50 m)** | **Satellite Imagery (100 m)** | **Hemispherical Photography** |
| Riverine | CAH | Peridomestic | 3.00% | 27.60% | 7.87% |
| | | Chacra | 42.80% | 50.80% | 7.39% |
| | | Forest | 100.00% | 98.20% | 93.57% |
| | NPR | Peridomestic | 83.40% | 91.30% | 43.85% |
| | | Chacra | 92.10% | 97.90% | 52.02% |
| | | Forest | 100.00% | 100.00% | 88.68% |
| | SEM | Peridomestic | 27.80% | 50.20% | 28.24% |
| | | Chacra | 67.60% | 75.20% | 30.45% |
| | | Forest | 85.50% | 88.40% | 89.99% |
| Highway | DOR | Peridomestic | 30.80% | 27.60% | 10.72% |
| | | Chacra | 68.80% | 76.90% | 45.16% |
| | | Forest | 94.20% | 68.20% | 85.95% |
| | NHO | Peridomestic | 21.10% | 35.80% | 8.17% |
| | | Chacra | 27.80% | 41.70% | 23.95% |
| | | Forest | 90.20% | 95.20% | 79.40% |
| | TRI | Peridomestic | 6.60% | 8.60% | 1.07% |
| | | Chacra | 61.60% | 49.30%** | 33.59% |
| | | Forest | 82.20% | 78.00% | 89.48% |

**Cloud obstructing view, could not calculate accurately

50m

| | | peri | chacra | forest |
|---|---|---|---|---|
| Riverine | mean | 38.07% | 67.50% | 95.17% |
| | stdev | 0.41172 | 0.2465 | 0.08372 |
| | 95CId | 0.80696 | 0.48314 | 0.16408 |
| Highway | mean | 19.50% | 52.73% | 88.87% |
| | stdev | 0.12179 | 0.21891 | 0.0611 |
| | 95CI | 0.23871 | 0.42906 | 0.11976 |

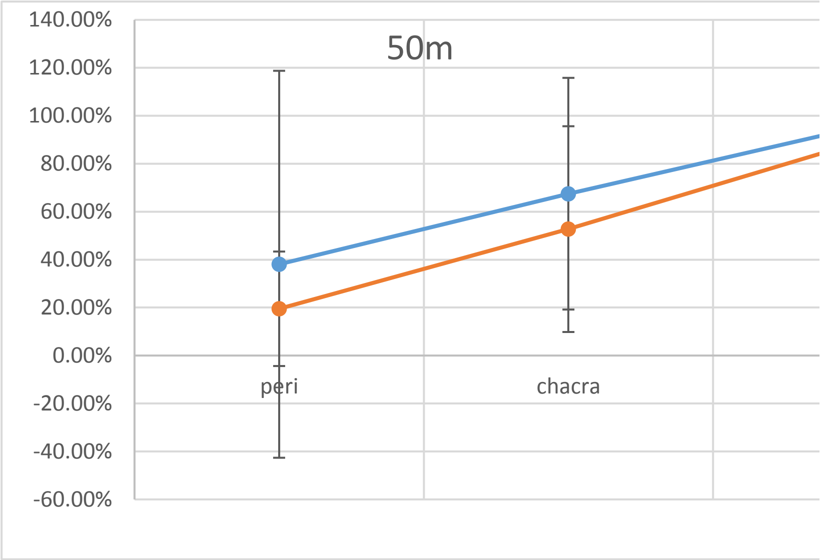


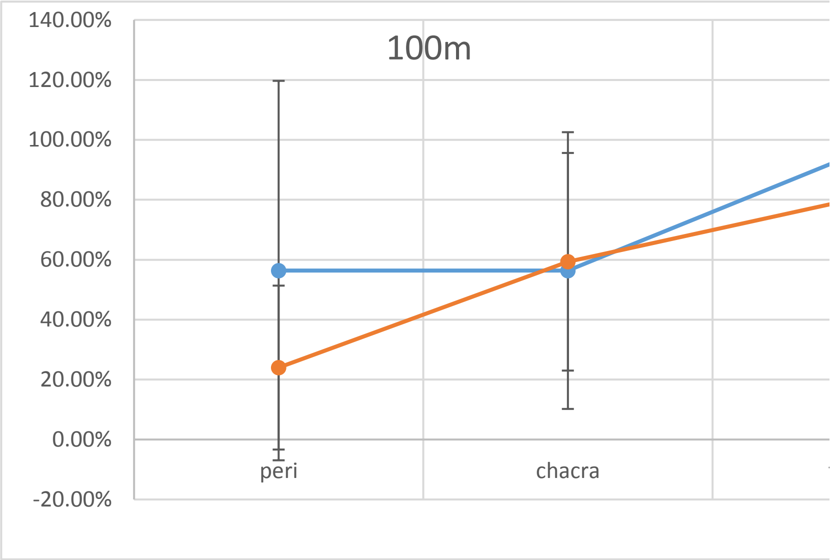

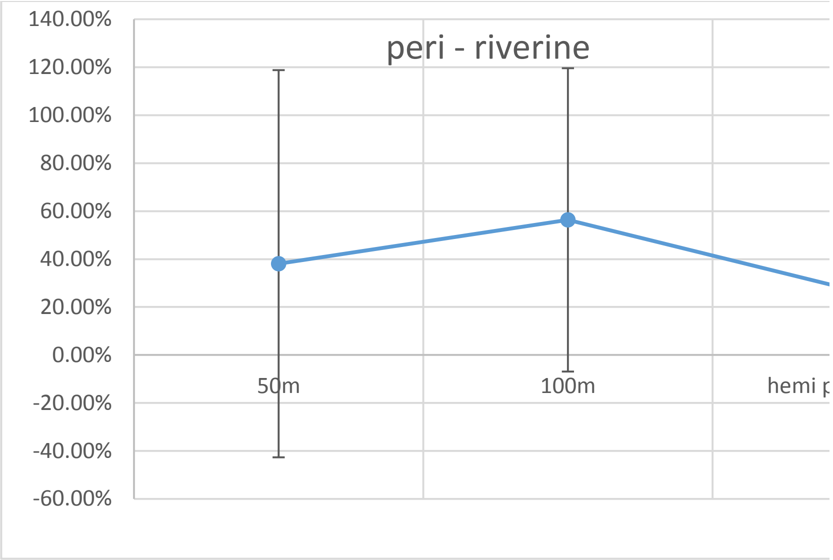
peri - riverine
140.00%
120.00%
100.00%
80.00%
60.00%
40.00%
20.00%
0.00%
-20.00%
-40.00%
-60.00%
50m
100m
hemi

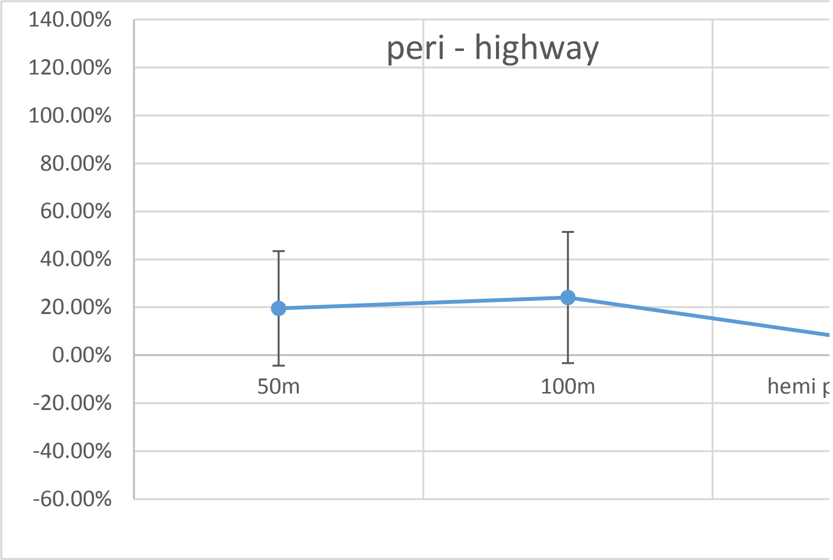
peri - highway
140.00%
120.00%
100.00%
80.00%
60.00%
40.00%
20.00%
0.00%
-20.00%
-40.00%
-60.00%
50m
100m
hemi

| 100m | | | | hemi photography | | |
|---|---|---|---|---|---|---|
| peri | chacra | forest | | peri | chacra | forest |
| 56.37% | 56.37% | 95.53% | | 26.65% | 29.95% | 90.75% |
| 0.32295 | 0.23555 | 0.06243 | | 0.18042 | 0.22319 | 0.02531 |
| 0.63297 | 0.46168 | 0.12236 | | 0.35363 | 0.43746 | 0.04961 |
| 24.00% | 59.30% | 80.47% | | 6.65% | 34.23% | 84.94% |
| 0.13953 | 0.18523 | 0.13668 | | 0.05001 | 0.1062 | 0.05115 |
| 0.27347 | 0.36305 | 0.26789 | | 0.09801 | 0.20814 | 0.10025 |

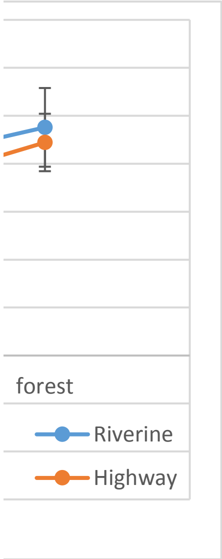


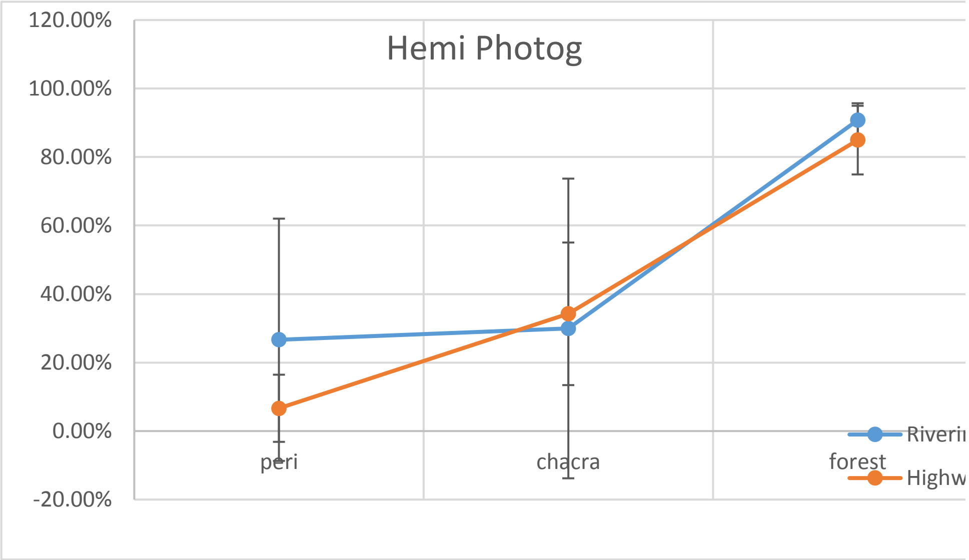


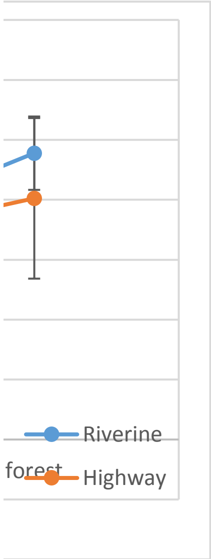

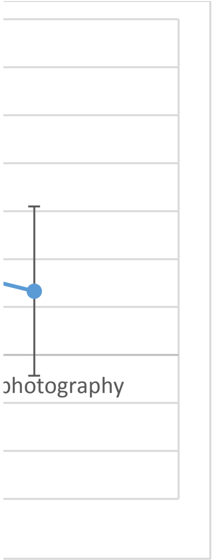

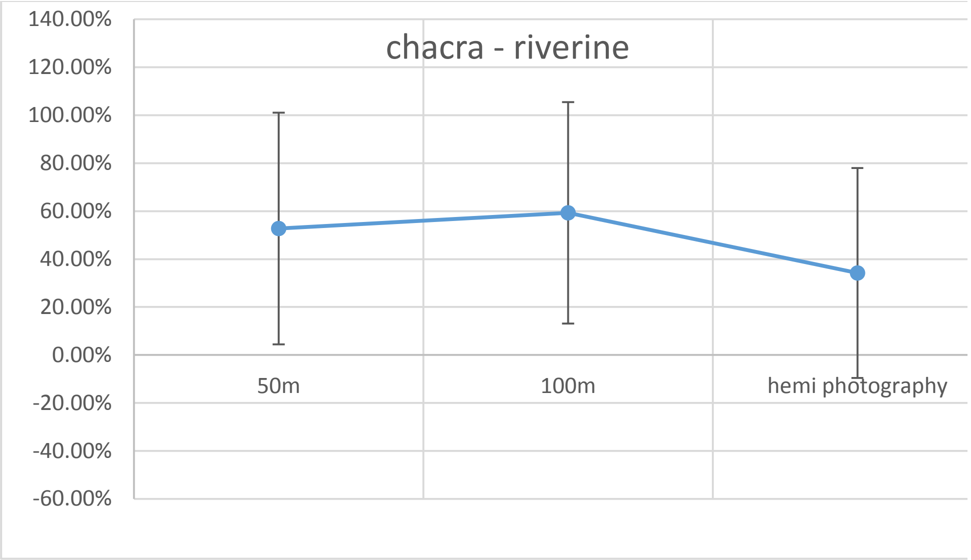
chacra - riverine
140.00%
120.00%
100.00%
80.00%
60.00%
40.00%
20.00%
0.00%
-20.00%
-40.00%
-60.00%
50m
100m
hemi photography

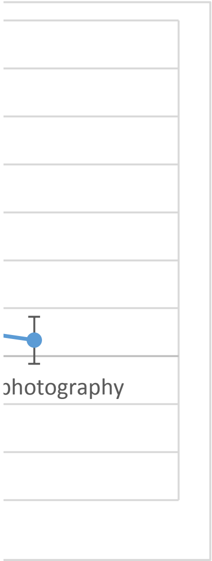

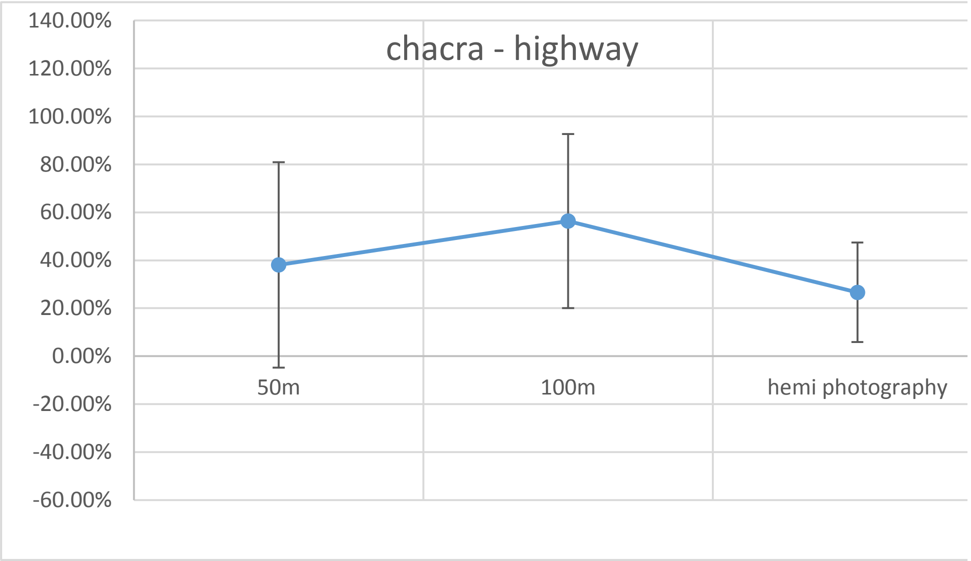
chacra - highway
140.00%
120.00%
100.00%
80.00%
60.00%
40.00%
20.00%
0.00%
-20.00%
-40.00%
-60.00%
50m
100m
hemi photography

ne
vay

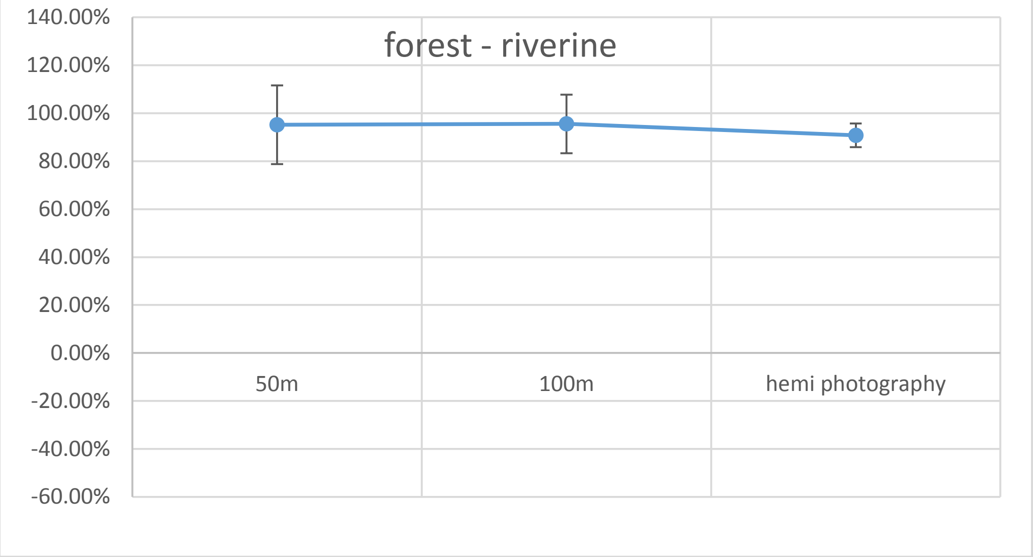
forest - riverine
140.00%
120.00%
100.00%
80.00%
60.00%
40.00%
20.00%
0.00%
-20.00%
-40.00%
-60.00%
50m
100m
hemi photography

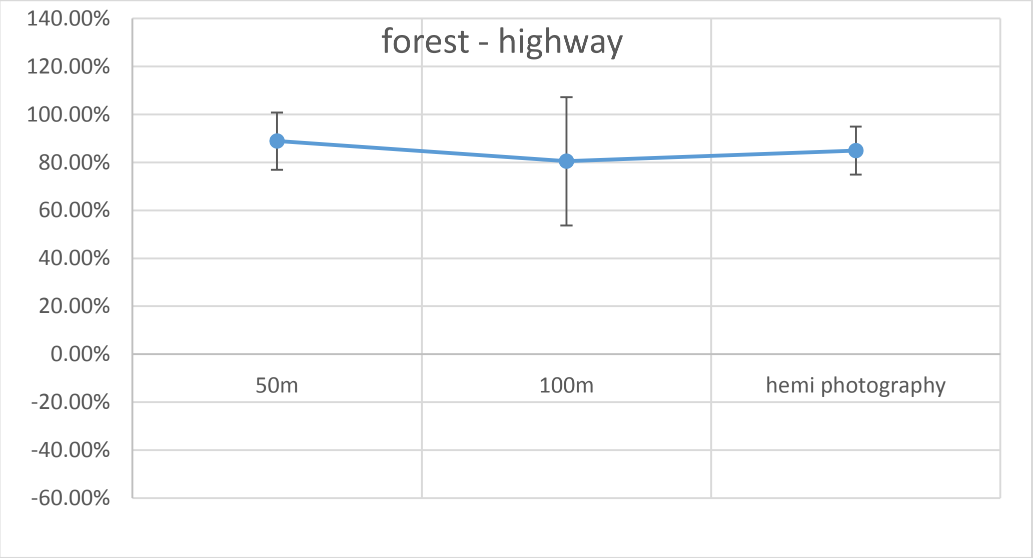
forest - highway
140.00%
120.00%
100.00%
80.00%
60.00%
40.00%
20.00%
0.00%
-20.00%
-40.00%
-60.00%
50m
100m
hemi photography
