# Supplementary material for: Evidence for temporal population replacement and the signature of ecological adaptation in a major Neotropical malaria vector in Amazonian Peru
Source: Malar J. 2015 Sep 29;14:375. doi: 10.1186/s12936-015-0863-4 (PMC4587789; doi:10.1186/s12936-015-0863-4)
Supplement: Supplementary file 7 — 10.1186/s12936-015-0863-4 Number of malaria cases (Plasmodium vivax and Plasmodium falciparum) in all of Peru (solid line, triangle markers) versus Loreto department (dashed line, square markers) [36]. Timing of PAMAFRO long lasting insecticidal net distribution (LLIN; red solid line) and El Niño event (blue solid line) shown [98, 100]. [file 12936_2015_863_MOESM7_ESM.pdf]

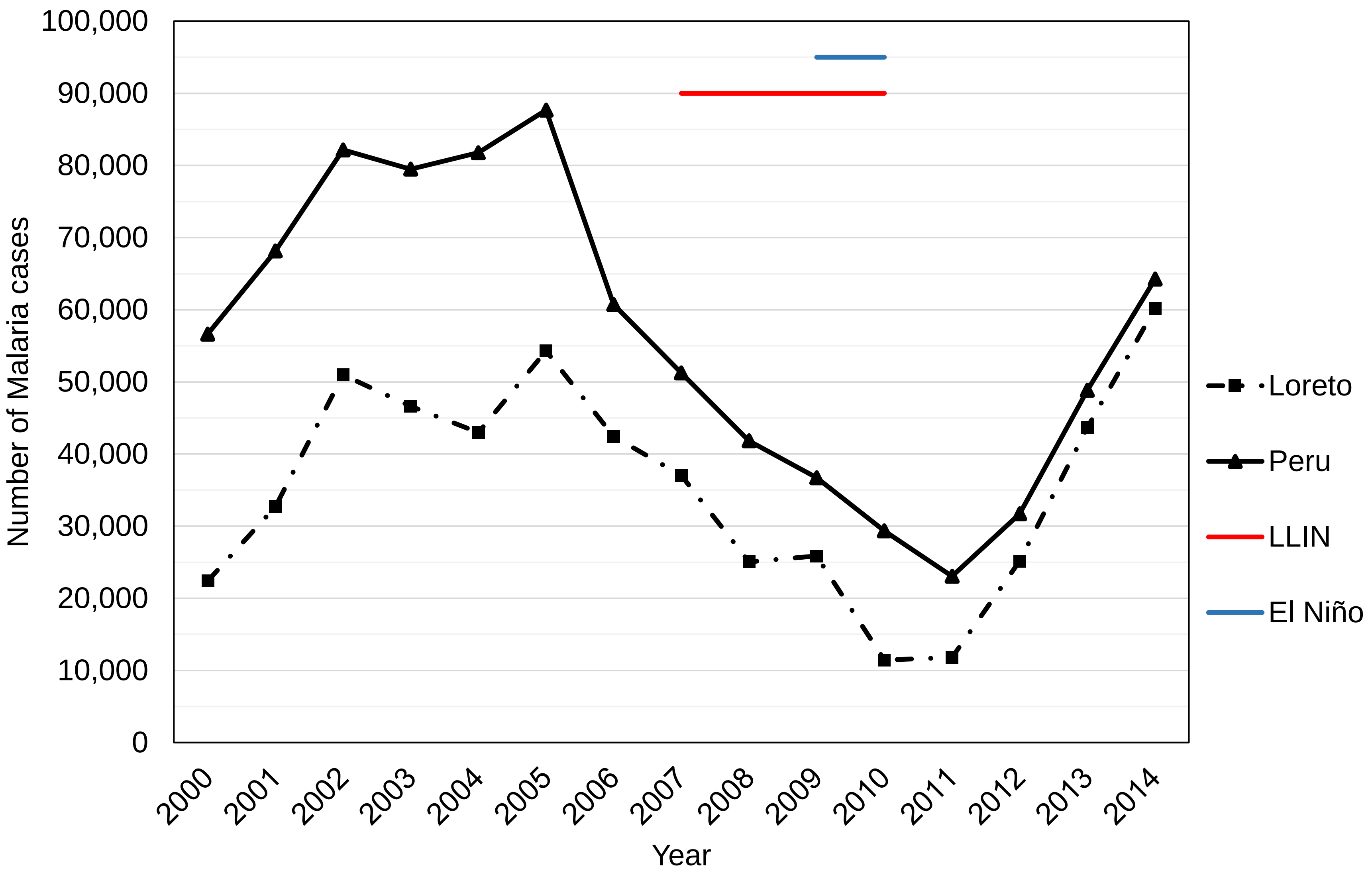

Additional file 7. Number of malaria cases (*Plasmodium vivax* and *Plasmodium falciparum*) in all of Peru (solid line, triangle markers) versus Loreto department (dashed line, square markers) (RENACE 2014). Timing of PAMAFRO long lasting insecticidal net distribution (LLIN; red solid line) and El Niño event (blue solid line) shown (NWS; OAS 2011).
